# TET2 is required to suppress mTORC1 signaling through urea cycle with therapeutic potential

**DOI:** 10.1038/s41421-023-00567-7

**Published:** 2023-08-08

**Authors:** Jing He, Mingen Lin, Xinchao Zhang, Ruonan Zhang, Tongguan Tian, Yuefan Zhou, Wenjing Dong, Yajing Yang, Xue Sun, Yue Dai, Yue Xu, Zhenru Zhang, Ming Xu, Qun-Ying Lei, Yanping Xu, Lei Lv

**Affiliations:** 1 MOE Key Laboratory of Metabolism and Molecular Medicine, Department of Biochemistry and Molecular Biology, School of Basic Medical Sciences, Fudan University, Shanghai, China; 2 Tongji Hospital, Shanghai Key Laboratory of Signaling and Disease Research, Frontier Science Center for Stem Cell Research, School of Life Sciences and Technology, Tongji University, Shanghai, China; 3 Shanghai Key Laboratory of Maternal Fetal Medicine, Clinical and Translational Research Center of Shanghai First Maternity and Infant Hospital, Tongji University, Shanghai, China; 4 UConn Center on Aging, UConn Health, Farmington, CT USA; 5 Fudan University Shanghai Cancer Center and Cancer Metabolism Laboratory, Institutes of Biomedical Sciences, Shanghai Medical College, Fudan University, Shanghai, China

**Keywords:** Cancer metabolism, RNA decay

## Abstract

Tumor development, involving both cell growth (mass accumulation) and cell proliferation, is a complex process governed by the interplay of multiple signaling pathways. TET2 mainly functions as a DNA dioxygenase, which modulates gene expression and biological functions via oxidation of 5mC in DNA, yet whether it plays a role in regulating cell growth remains unknown. Here we show that TET2 suppresses mTORC1 signaling, a major growth controller, to inhibit cell growth and promote autophagy. Mechanistically, TET2 functions as a 5mC “eraser” by mRNA oxidation, abolishes YBX1–HuR binding and promotes decay of urea cycle enzyme mRNAs, thus negatively regulating urea cycle and arginine production, which suppresses mTORC1 signaling. Therefore, TET2-deficient tumor cells are more sensitive to mTORC1 inhibition. Our results uncover a novel function for TET2 in suppressing mTORC1 signaling and inhibiting cell growth, linking TET2-mediated mRNA oxidation to cell metabolism and cell growth control. These findings demonstrate the potential of mTORC1 inhibition as a possible treatment for TET2-deficient tumors.

## Introduction

The ten-eleven translocation enzyme TET2 is a DNA dioxygenase, which modulates gene expression by catalyzing the conversion of 5-methylcytosine (5mC) to 5-hydroxymethylcytosine (5hmC), then to 5-formylcytosine and 5-carboxylcytosine (5caC)^1,2^. 5caC is then demethylated to cytosine via the action of thymine DNA glycosylase^3,4^. Besides being an intermediate during demethylation, existing data indicate that 5hmC per se is an epigenetic mark critical for various biological and pathological processes^5,6^ and can be utilized for assessing the efficacy of patients’ response to anti-PD-1/PD-L1 immunotherapy^7^. Notably, 5hmC level is significantly reduced across different types of tumors and inversely correlates with tumor cell proliferation^8^. As an epigenetic modifier, TET2 has fundamental roles in cell fate determination^9,10^, cell differentiation^11,12^ and tumor development^13–19^. Moreover, TET2 is also involved in mRNA stability regulation via inducing its oxidation^20^. These findings suggest diverse functions of TET2 in physiological and pathological processes.

TET2 is a tumor suppressor and loss-of-function mutations of TET2 frequently happen in hematopoietic malignancy^12,21^. Interestingly, a subset of acute myeloid leukemia and glioma patients without TET2 mutations bear isocitrate dehydrogenases 1 and 2 (IDH1/2) mutations, which produce D-2-hydroxyglutarate to competitively inhibit TET2 activity^22,23^. In addition to mutations, TET2 activity is significantly suppressed in multiple tumors by different mechanisms^24–26^. Consistently, restoration of TET2 activity blocks aberrant self-renewal and leukemia progression, further supporting the vital role of TET2 in suppressing tumor development.

The mammalian target of rapamycin (mTOR) is an evolutionarily conserved serine/threonine protein kinase that serves as a central hub of metabolic signaling and cell growth control via coordinating diverse sets of environmental inputs such as growth factors and nutrients^27^. The activation of mammalian target of rapamycin complex 1 (mTORC1) includes two steps, lysosomal translocation and activation in lysosome, which is dominantly regulated by amino acids and TSC-Rheb axis, respectively^28^. Energy, oxygen, growth factors or insulin activates mTORC1 mainly through TSC-Rheb axis, while lysosomal translocation is dominantly triggered by amino acids via manipulating the interactions between amino acid sensors and mTORC1 upstream regulators^28^. mTORC1 senses cytosolic and intra-lysosomal amino acids through distinct mechanisms. Cytosolic arginine, leucine or methionine can disrupt the association between its sensor and GATOR2 or GATOR1 to facilitate the translocation of mTORC1 to lysosome by Rag dimer^27^, while glutamine promotes mTORC1 lysosomal translocation in a Rag-independent manner^29^. SLC38A9, the lysosomal amino acid transporter, is required for arginine-mediated mTORC1 activation by collaborating with Rag–Ragulator–v-ATPase complex^27^. Besides, amino acid starvation could compromise mTORC1 activity induced by Rheb GTPase^30^, highlighting the relevance of amino acids for mTORC1 activation.

Here, we reported that TET2 modulates cellular arginine concentration through urea cycle by mRNA oxidation and suppresses mTORC1 signaling, thus inhibiting tumor cell growth. Moreover, deficiency of TET2 sensitizes tumors to mTORC1 inhibitors.

## Results

### Deficiency of Tet2 activates mTORC1 signaling

**Fig. 1:**
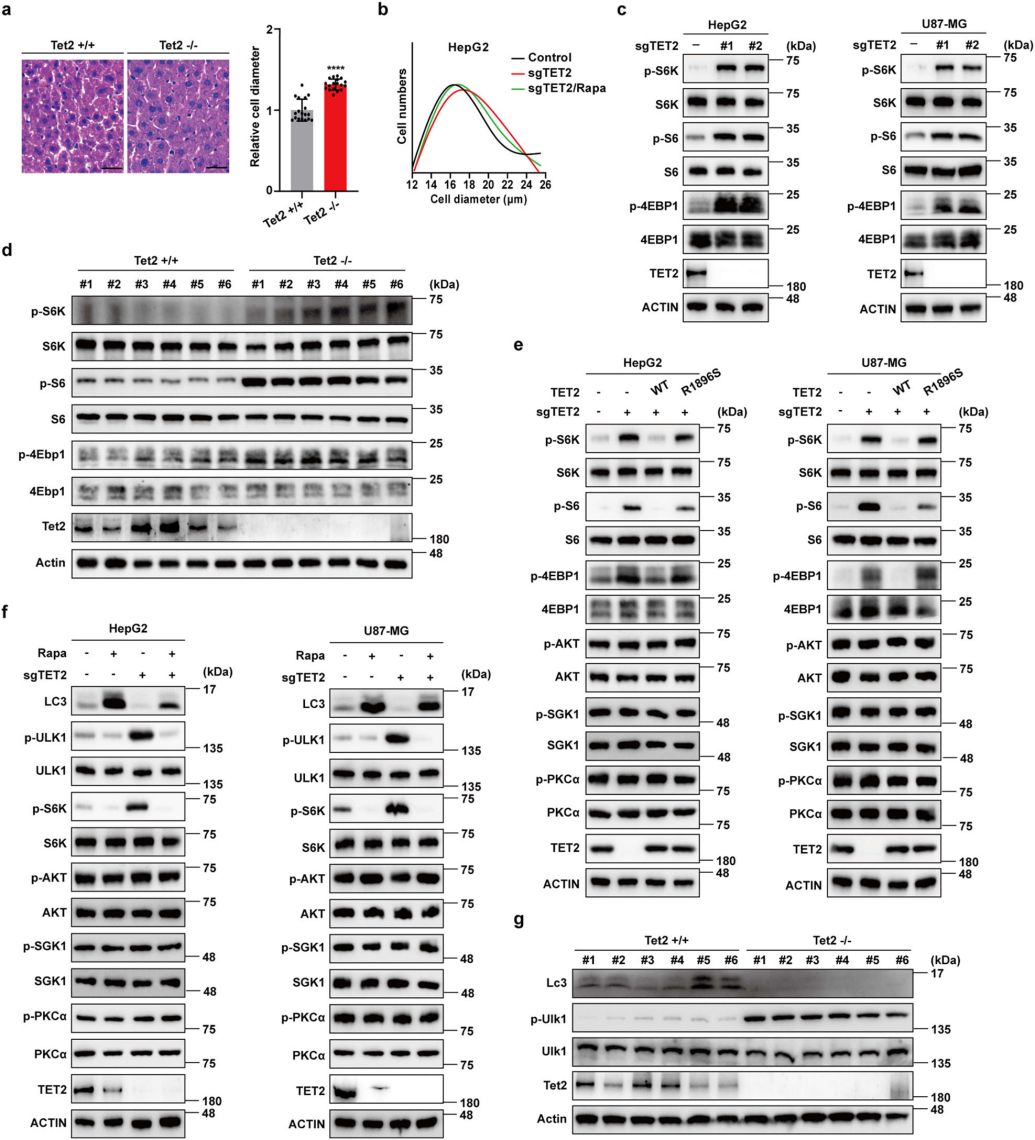
Deficiency of Tet2 activates mTORC1 signaling and inhibits autophagy. **a** Deficiency of Tet2 increases cell size of mouse liver cells. Left panel, wild-type (*Tet2*^+/+^, WT) and *Tet2* knockout (*Tet2*^–/–^, KO) mice livers were subjected to HE staining. Scale bars, 25 μm. Right panel, cell diameter was calculated by ImageJ. *n* = 6 biologically independent animals per group. **b** *TET2* KO increases cell size of tumor cells, which can be rescued by rapamycin. Cells were treated with 20 nM rapamycin for 72 h. **c** *TET2* KO increases phosphorylation levels of S6K, S6 and 4EBP1 in tumor cells. **d** Deficiency of Tet2 leads to increased phosphorylation levels of S6K, S6 and 4Ebp1 in mice livers. *n* = 6 biologically independent animals per group. **e** Re-introduction of WT TET2, but not catalytic mutant TET2 (R1896S), can block mTORC1 activation induced by TET2 deficiency. **f** *TET2* KO reduces autophagy in tumor cells, which can be rescued by rapamycin. Cells were treated with 10 nM rapamycin for 24 h. **g** Tet2 is required for activation of autophagy in mouse livers. Autophagy activation in mouse livers was determined by western blot. *n* = 6 biologically independent animals per group.

Tumor development involves both cell growth (cell size) and proliferation, while cell growth is required for proliferation. TET2 is well established to repress cell proliferation^24,31^, yet its function on cell growth remains unclear. We hypothesized that Tet2 may have a role in cell growth. To verify this hypothesis, we first compared the liver cell size of wild-type (*Tet2*^+/+^, WT) mice and *Tet2* knockout (*Tet2*^–/–^, KO) mice. Strikingly, as shown in Fig. 1a, Tet2 deficiency significantly increases the size of mouse liver cells (Fig. 1a). To confirm this finding, we performed sgRNA-mediated knockout of *TET2* in tumor cells. Consistently, deletion of TET2, but not TET1 or TET3, also increases the size of tumor cells (Supplementary Fig. S1a, b). Cell size is often coupled with cell cycle and polyploidy, we thus examined whether cell cycle and polyploidy are aberrantly affected by TET2 deletion and found that the percentage of cells in G2/M (Supplementary Fig. S1c) and polyploidy phases (Supplementary Fig. S1d) are increased in *TET2* KO cells and primary hepatocytes of *Tet2* KO mice, respectively. Since it is well established that mTORC1 is the major regulator of cell size^27^, we tested the effect of its inhibitor rapamycin on the phenotype of *TET2* KO cells. Results showed that rapamycin treatment reverses the increased cell size induced by *TET2* KO (Fig. 1b), suggesting mTORC1 may function at the downstream of TET2. Consistently, knockout of *TET2*, but not *TET1* or *TET3*, dramatically increases phosphorylation levels of S6K (T389), S6 (S235/236) and 4EBP1 (T37/46) (Fig. 1c, d and Supplementary Fig. S1e–g), demonstrating that TET2 deficiency promotes mTORC1 activation. Moreover, we examined the mRNA levels of 30 mTORC1 target genes involved in glycolysis, OXPHOS, pentose phosphate pathway, fatty acid biosynthesis, ribosome and lysosome biogenesis in *TET2* KO cells. Interestingly, all TET2-mediated regulation of mTORC1 target genes can be rescued by rapamycin treatment (Supplementary Fig. S1h), further demonstrating that TET2 suppresses mTORC1 signaling. We next examined whether the catalytic activity of TET2 is required for its regulation on cell size and mTORC1 signaling. Compared to wild-type TET2, catalytic mutant (R1896S) almost has no effect on both cell size (Supplementary Fig. S1i) and mTORC1 signaling (Fig. 1e). Since mTORC1 is a vital negative regulator of autophagy^32^, we checked autophagy-related markers and found the reduced LC3 II/I ratio, while elevated level of phosphorylation of ULK1 in TET2-deficient tumor cells, both of which can be rescued by rapamycin treatment (Fig. 1f), indicating that TET2 is also required for activation of autophagy. Unlike the increased phosphorylation of Ulk1, both LCI and LCII diminish dramatically in Tet2-deficient mouse livers (Fig. 1g), which is confirmed in *TET2* KO tumor cells (Supplementary Fig. S1j), suggesting that Tet2 might promote activation of autophagy in both mTORC1-dependent and -independent manners. Together, these findings suggest that *TET2* KO activates mTORC1 signaling.

### TET2 is a negative regulator of urea cycle

**Fig. 2:**
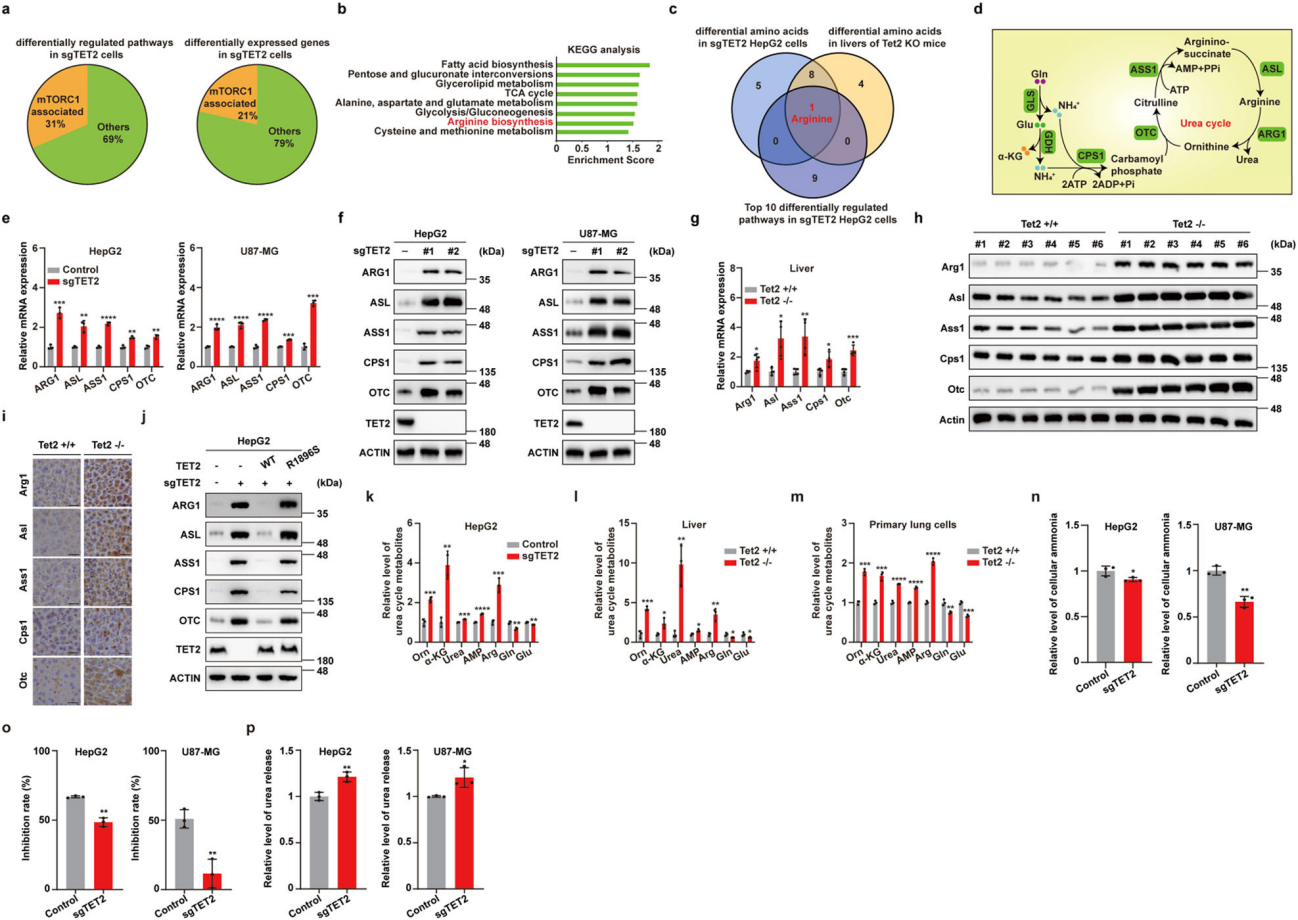
TET2 is a negative regulator of urea cycle. **a** Approximately 31% and 21% of differentially regulated pathways and expressed genes in *sgTET2* cells associate with mTORC1, respectively. RNA-seq of WT and *TET2* KO HepG2 cells was performed. *n* = 3 biologically independent samples per group. **b** KEGG analysis of metabolic-related pathways of RNA-seq data from WT and *TET2* KO HepG2 cells. *n* = 3 biologically independent samples per group. **c** Combinational analysis of RNA-seq data and differential amino acids in *sgTET2* HepG2 cells and livers of *Tet2* KO mouse. **d** Schematic description of arginine synthesis through urea cycle. **e** *TET2* KO increases mRNA levels of urea cycle enzymes in tumor cells. *n* = 3 biologically independent samples per group. **f** *TET2* KO increases protein levels of urea cycle enzymes in tumor cells as indicated. **g** Deficiency of Tet2 increases mRNA levels of urea cycle enzymes in mouse livers. *n* = 4 biologically independent animals per group. **h** Deficiency of Tet2 increases protein levels of urea cycle enzymes in mouse livers. The protein levels of the enzymes were determined by western blot. *n* = 6 biologically independent animals per group. **i** Higher expression levels of urea cycle enzymes in *Tet2* KO mouse livers compared to WT mouse. Expression levels of urea cycle enzymes in mouse livers were determined by IHC analysis. Scale bars, 25 μm. *n* = 6 biologically independent animals per group. **j** Re-introduction of WT TET2, but not catalytic mutant TET2 (R1896S), can rescue upregulation of urea cycle enzymes induced by TET2 deficiency. **k** Deficiency of TET2 increases metabolites of urea cycle in HepG2 cells. *n* = 2–3 biologically independent samples per group. **l** Deficiency of Tet2 increases levels of urea cycle metabolites in mouse livers. *n* = 3 biologically independent animals per group. **m** Deficiency of Tet2 increases levels of urea cycle metabolites in primary lung cells. *n* = 3 biologically independent samples per group. **n** Ammonia, a substrate of urea cycle, is more rapidly consumed by *TET2* KO tumor cells. Cells were treated with 10 mM NH_4_Cl for 24 h and cellular ammonia levels were determined. *n* = 3 biologically independent samples per group. **o** Cell growth inhibition caused by exogenous ammonia treatment is relieved in *TET2* KO tumor cells. Cells were treated with 10 mM NH_4_Cl for 24 h and cell growth was determined by CCK-8 kit. *n* = 3 biologically independent samples per group. **p** Urea release is increased in *TET2* KO tumor cells. Cells were treated with 10 mM NH_4_Cl for 24 h and urea levels were determined. *n* = 3 biologically independent samples per group.

AKT is an upstream regulator of mTORC1 signaling pathway^33^, so we first determined whether TET2 deficiency affects AKT activity. There is almost no change of AKT phosphorylation in *TET2* KO cells (Supplementary Fig. S2a), suggesting that the suppression of mTORC1 activity by TET2 is independent of AKT. To reveal the underlying mechanism of *TET2* KO-induced mTORC1 activation, we performed RNA-seq analysis for WT and *TET2* KO HepG2 cells and found that approximately 31% and 21% of differentially regulated pathways and genes in *TET2* KO cells associate with mTORC1, respectively (Fig. 2a). Furthermore, Kyoto Encyclopedia of Genes and Genomes (KEGG) analysis of metabolic-related pathways revealed that a variety of mTORC1 upstream and downstream pathways are aberrantly regulated in *TET2* KO cells (Fig. 2b and Supplementary Fig. S2b), supporting the regulation of TET2 on mTORC1. Interestingly, arginine biosynthesis is identified as one of the top upregulated gene sets in TET2-deficient cells (Fig. 2b), since arginine is a potent activator of mTORC1^34^. As amino acids are vital for mTORC1 activation, we thus examined the effect of *TET2* KO on the levels of 20 amino acids by mass spectrometry and found that levels of several amino acids including arginine are altered in both *TET2* KO tumor cells and mouse livers (Supplementary Fig. S2c, d). Combinational analysis of RNA-seq data and differential amino acids revealed that arginine is the unique amino acid identified in all three datasets (Fig. 2c). Elevation of other amino acids may be attributed to upregulation of several transporters upon deletion of TET2 (Supplementary Fig. S2e). Since urea cycle is the main source of arginine biosynthesis in cells^35^ (Fig. 2d), we then investigated whether urea cycle is the mediator between TET2 deficiency and mTORC1 activation. Surprisingly, both mRNA and protein levels of several urea cycle enzymes (ARG1, ASL, ASS1, CPS1 and OTC) are upregulated in *TET2* KO cells (Fig. 2e, f and Supplementary Fig. S2f) and *Tet2* KO mouse livers (Fig. 2g, h). Immunohistochemistry (IHC) staining results showed that protein levels of these urea cycle enzymes are also elevated in *Tet2* KO mouse livers (Fig. 2i). Re-introduction of wild-type TET2, but not catalytic mutant (R1896S), could reverse the expression of urea cycle enzymes in *TET2* KO cells (Fig. 2j). Consistently, the metabolites of urea cycle, including arginine, are higher in TET2-deficient cells, livers and primary lung cells (Fig. 2k–m), supporting the TET2-urea cycle-mTORC1 hypothesis. Ammonia is a substrate of urea cycle and possesses growth inhibition effect in cells^35^. We found *TET2* KO promotes ammonia consumption in tumor cells (Fig. 2n), and the growth-inhibiting effect on tumor cells by exogenous ammonia can be relieved by knockout of *TET2* (Fig. 2o). Consistently, more urea, the end product of urea cycle, is released to extracellular surroundings from *TET2* KO cells (Fig. 2p). Taken together, these data suggest that TET2 is a negative regulator of urea cycle enzymes, ammonia utilization, and the production of arginine and urea.

### TET2 suppresses mTORC1 activation via urea cycle

**Fig. 3:**
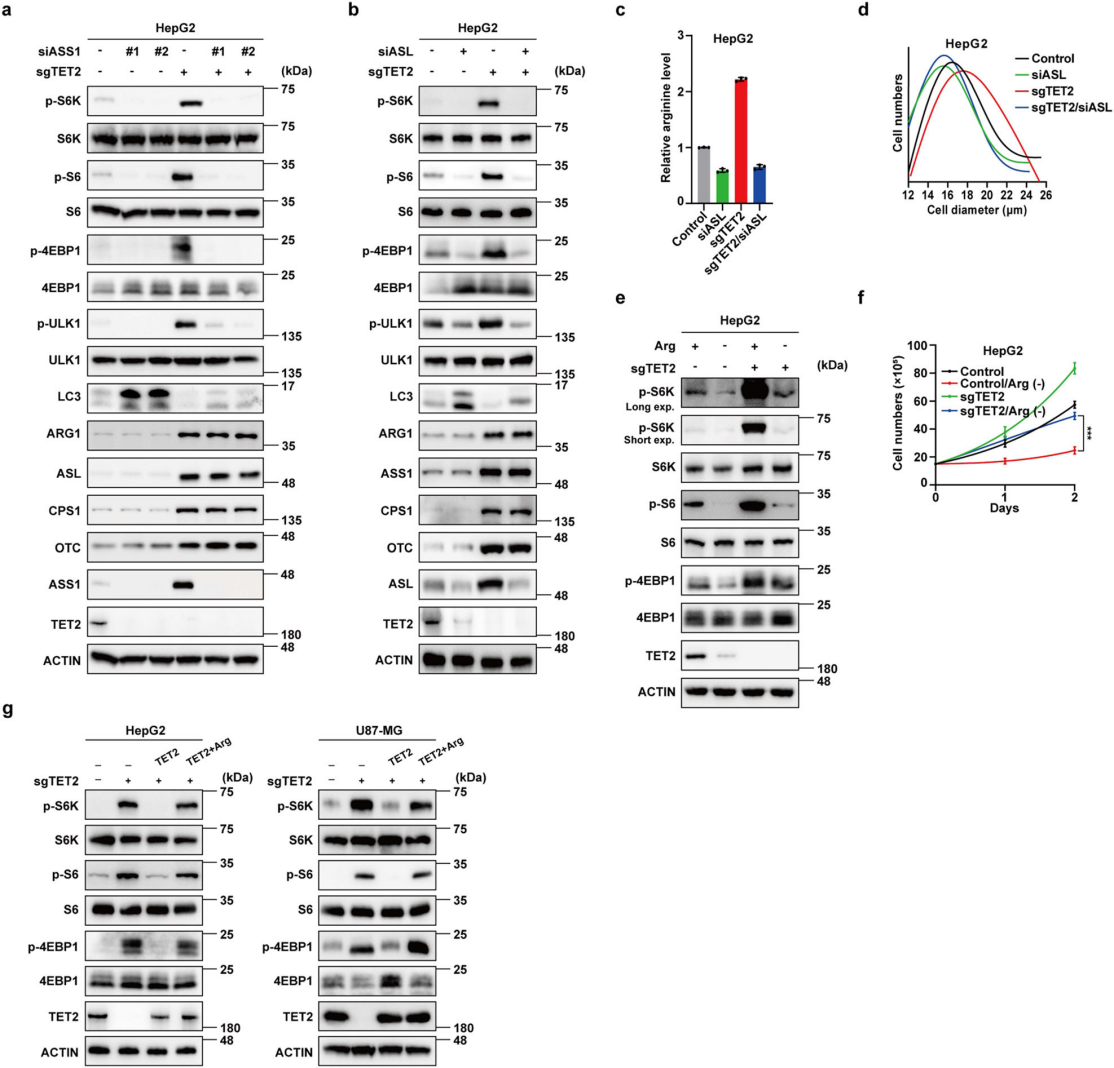
TET2 suppresses mTORC1 activation via urea cycle. **a** Knockdown of ASS1 blocks TET2 deletion-induced phosphorylation of S6K, S6 and 4EBP1. **b** Knockdown of ASL blocks TET2 deletion-induced phosphorylation of S6K, S6 and 4EBP1. **c** Knockdown of ASL blocks TET2 deletion-induced upregulation of arginine level. *n* = 3 biologically independent samples per group. **d** Knockdown of ASL blocks TET2 deletion-induced increase in cell size. *n* = 3 biologically independent samples per group. **e** *sgTET2* cells are more resistant to arginine starvation with higher mTORC1 activity. Long exp long exposure, Short exp short exposure. **f** *sgTET2* cells are more resistant to arginine starvation with higher proliferation ability. *n* = 3 biologically independent samples per group. **g** Arginine supplementation reverses the suppressive effect of TET2 overexpression on mTORC1 in *sgTET2* HepG2 cells.

Subsequently, we examined whether manipulation of urea cycle can regulate mTORC1. Results showed that both overexpression of arginine-producing enzymes (ASL or ASS1) and knockdown of arginine-consuming enzyme ARG1 enhance mTORC1 signaling and cellular arginine concentrations (Supplementary Fig. S3a–d). Consistently, overexpression of ASL increases cell size of HepG2 cells (Supplementary Fig. S3e). We next investigated whether *TET2* KO activates mTORC1 via urea cycle. Knockdown either ASL or ASS1 blocks *TET2* KO-induced upregulation of mTORC1 activity (Fig. 3a, b and Supplementary Fig. S3f, g), arginine level (Fig. 3c) and increase in cell size (Fig. 3d). Due to the enhanced capacity of arginine biosynthesis, *TET2* KO cells are more resistant to arginine starvation with higher mTORC1 activity (Fig. 3e), as well as proliferation ability (Fig. 3f). Furthermore, arginine supplementation reverses the suppressive effect of TET2 overexpression on mTORC1 in *TET2* KO cells (Fig. 3g). Collectively, these results strengthen our finding that TET2 suppresses mTORC1 signaling via downregulation of arginine biosynthesis. CASTOR1/2 and SLC38A9 function in parallel to sense cellular arginine and are involved in regulation of mTORC1 activity^36,37^. As a negative regulator, knockout of *CASTOR1* enhances mTORC1 activity both in WT and *TET2* KO cells (Supplementary Fig. S3h), while loss of SLC38A9, the positive regulator of mTORC1, abolishes mTORC1 activation caused by TET2 deficiency (Supplementary Fig. S3i).

Notably, TET2 protein levels are significantly declined upon knockdown of ASL or ASS1 (Fig. 3a, b and Supplementary Fig. S3f, g), suggesting that mTORC1 may exert a feedback regulation on TET2 expression. To test this hypothesis, we examined TET2 mRNA and protein levels after rapamycin treatment and found that rapamycin reduces TET2 protein level, but not mRNA level (Supplementary Fig. S3j, k). As arginine and glutamine are well-known upstream signals of mTORC1^29^, we checked the effects of arginine or glutamine starvation on TET2 expression and observed that arginine or glutamine depletion reduces TET2 protein level as well, while it has no effect at mRNA level (Supplementary Fig. S3l–o). Consistently, either arginine or glutamine stimulation dramatically increases expression of TET2 at protein level rather than mRNA level (Supplementary Fig. S3p, q). To distinguish whether TET2 deletion-induced elevation of ARG1, ASL, ASS1, CPS1 and OTC1 is a cause or result of mTORC1 activation, we treated *TET2* KO cells with rapamycin and monitored its effect on the expression of urea cycle enzymes and found that rapamycin treatment exhibits regulatory effect on mRNA instead of protein level of urea cycle enzymes (Supplementary Fig. S3r, s), suggesting that mTORC1 may be involved in translation of urea cycle enzymes and the effect caused by loss of TET2 is compromised by rapamycin, resulting in a stable protein level of urea cycle enzymes. Nevertheless, the mRNA level of *OTC* is suppressed by rapamycin independent of TET2. Compared with *TET2* KO cells, the metabolic background under rapamycin treatment is entirely divergent, suggesting that mTORC1 may regulate OTC expression via other pathways. mTORC1 has been demonstrated as a master regulator of cell growth through integrating intracellular and extracellular signals to promote protein translation^27^. Combined with our data, mTORC1 may modulate TET2 expression at protein level through translation stimulation and the mTORC1-TET2 axis might present a route for intracellular epigenetic regulation by multiple signals.

### TET2 restrains urea cycle through mRNA oxidation

**Fig. 4:**
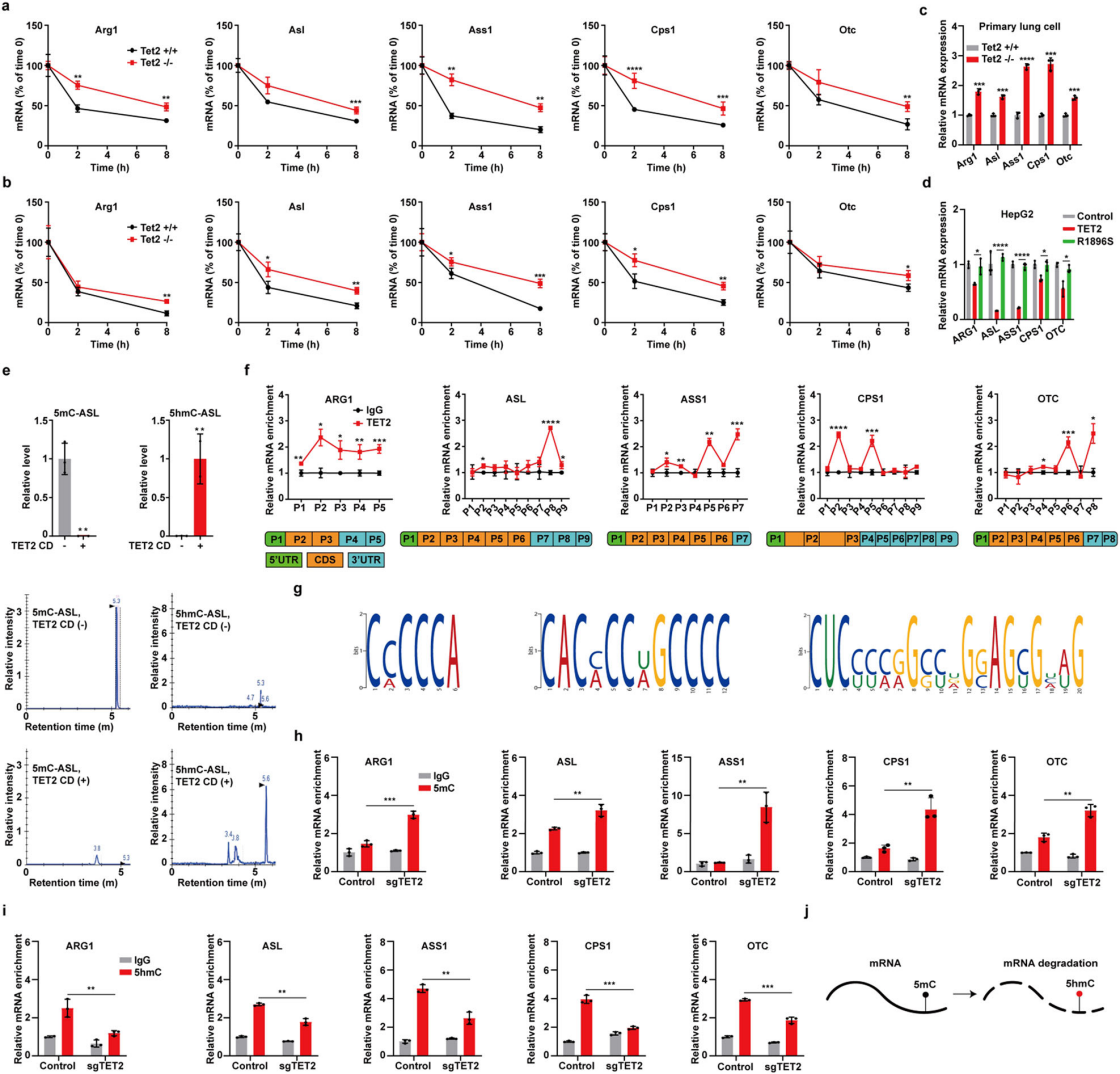
TET2 restrains urea cycle through mRNA oxidation. **a,** **b** *Tet2* KO increases mRNA stability of urea cycle enzymes. Primary liver cells (**a**) and primary lung cells (**b**) from WT and *Tet2* KO mice were treated with 5 μg/mL actinomycin D for different time points as indicated. mRNA decay of urea cycle enzymes was quantified by qPCR. *n* = 3 biologically independent animals per group. **c** *Tet2* KO increases mRNA levels of urea cycle enzymes in primary lung cells. *n* = 3 biologically independent animals per group. **d** Catalytic activity of TET2 is required to induce mRNA decay of urea cycle enzymes. WT and catalytic mutant (R1896S) TET2 were transfected into HepG2 cells. mRNA levels of urea cycle enzymes were quantified by qPCR. *n* = 3 biologically independent samples per group. **e** LC-MS quantifying 5mC and its oxide 5hmC levels in methylated RNA in the absence or presence of TET2 catalytic domain (CD) in vitro. 5mC at 5.3 min, 5hmC at 5.6 min. *n* = 2–3 biologically independent samples per group. **f** TET2 associates with endogenous 3′ UTR and CDS of urea cycle enzyme mRNAs. TET2 binding sites were mapped in the mRNA of urea cycle enzymes by qPCR of TET2 RIP product. RIP, RNA immunoprecipitation assay. *n* = 2–3 biologically independent samples per group. **g** Conserved motifs in 3′ UTR of five urea cycle enzyme mRNAs. **h** *TET2* KO increases mRNA 5mC levels of urea cycle enzymes. mRNA of urea cycle enzymes was immune-precipitated from control and *TET2* KO HepG2 cells with anti-5mC antibody and quantified by qPCR. *n* = 3 biologically independent samples per group. **i** *TET2* KO reduces mRNA 5hmC levels of urea cycle enzymes. mRNA of urea cycle enzymes was immune-precipitated from control and *TET2* KO HepG2 cells with anti-5hmC antibody and quantified by qPCR. *n* = 2–3 biologically independent samples per group. **j** TET2 promotes mRNA decay via mediating its oxidation from 5mC to 5hmC.

We then investigated the mechanism whereby TET2 inhibits urea cycle. To find out whether TET2 deficiency-induced dysregulation of DNA methylation affects the expression of urea cycle enzymes, we conducted whole genome bisulfite sequencing in *TET2* KO cells. KEGG enrichment analysis revealed that methylation levels of numerous genes involved in multiple biological pathways are aberrantly regulated in *TET2* KO cells (Supplementary Fig. S4a). Data analysis demonstrated that the differentially methylated regions of whole genome regulated by TET2 are mainly enriched in introns, CpG island (CGI) shores, promoters, exons, transcription start site regions and CGIs (Supplementary Fig. S4b). There are a large number of genes whose methylation levels are increased in various regions in *TET2* KO cells compared to WT cells (Supplementary Fig. S4c–i), while methylation levels across gene bodies of *ARG1*, *ASL*, *ASS1* and *OTC*, and promoter regions of *ARG1*, *ASL* and *CPS1* are not significantly changed; of note, methylation levels in gene body of *CPS1* are decreased in *TET2* KO cells, and the methylation levels in promoters of *ASS1* and *OTC* are hard to determine, as there are few CpG in their promoters (Supplementary Fig. S4j–q), suggesting DNA methylation is not involved in TET2 deficiency-mediated regulation of urea cycle enzymes. TET2 has been reported to reduce mRNA stability via oxidizing mRNA 5mC to 5hmC^20^, which led us to examine whether TET2 suppresses expression of urea cycle enzymes via mRNA oxidation. We found that loss of TET2 indeed leads to increased mRNA stability of urea cycle enzymes in tumor cells (Supplementary Fig. S5a, b) and mouse primary liver (Fig. 4a) and lung cells (Fig. 4b) when the de novo transcription has been blocked. Consistently, the expression of urea cycle enzymes is increased in primary lung cells upon deletion of Tet2 (Fig. 4c). To determine whether TET2 exerts a global effect on mRNA stability, we examined the effect of TET2 on other mRNAs encoding housekeeping gene (*GAPDH*) and differentially expressed genes (*TNS4* and *ALB*) in *TET2* KO cells (Supplementary Fig. S5c). Results showed that TET2 has no effect or exhibits a minor effect on the mRNA stability of these three genes (Supplementary Fig. S5d), indicating that TET2-mediated regulation of mRNA stability is gene-specific. Moreover, overexpression of wild-type TET2 rather than catalytic mutant decreases mRNA levels of urea cycle enzymes (Fig. 4d and Supplementary Fig. S5e), demonstrating that TET2-mediated mRNA stability regulation of urea cycle enzymes is dependent on its catalytic activity. To provide direct evidence for TET2-induced mRNA decay of urea cycle enzyme mRNAs, we synthesized *ASL* mRNA containing 5mC for in vitro assay. As shown in Fig. 4e and Supplementary Fig. S5f, 5mC in *ASL* mRNA is oxidized to 5hmC by TET2 catalytic domain (CD) (Fig. 4e and Supplementary Fig. S5f). Subsequently, in vivo TET2 binding sites were mapped in the mRNAs of urea cycle enzymes by qPCR of TET2 RNA immunoprecipitation (RIP) product, which demonstrated that TET2 associates with endogenous 3′ UTR and CDS of urea cycle enzyme mRNAs (Fig. 4f). Interestingly, all the 3′ UTRs of urea cycle enzyme mRNAs contain three conserved motifs with multiple cytosines (Fig. 4g). Furthermore, *TET2* KO increases 5mC levels (Fig. 4h) and reduces 5hmC levels (Fig. 4i) of these urea cycle enzyme mRNAs, while exerts no effect on mRNAs of *GAPDH*, *TNS4* and *ALB* (Supplementary Fig. S5g, h). These data support the model that TET2 promotes mRNA decay of urea cycle enzymes via mRNA oxidation (Fig. 4j).

### TET2 destabilizes mRNA by abolishing the function of YBX1 and HuR

**Fig. 5:**
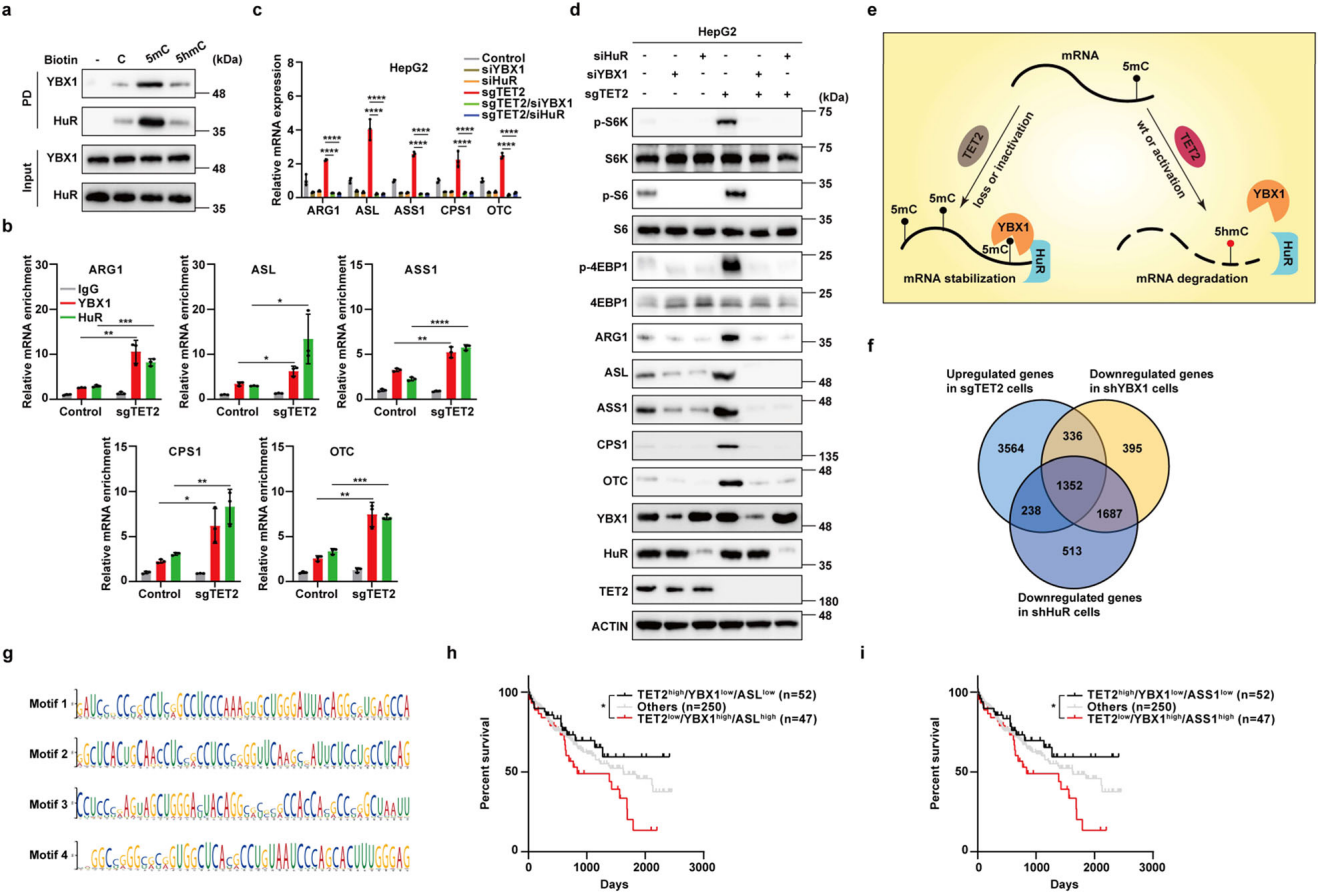
TET2 destabilizes mRNA by abolishing the function of YBX1 and HuR. **a** TET2-mediated oxidation of mRNA 5mC disrupts its binding with YBX1 and HuR. Pull-down assay was performed by incubating C, 5mC and 5hmC oligos of *ASL* mRNA with cell lysate from HepG2 cells. 5mC oligos were catalyzed to 5hmC by TET2 in vitro. **b** TET2 deficiency promotes YBX1 and HuR binding to mRNAs of urea cycle enzymes. *n* = 3 biologically independent samples per group. **c** Knockdown of YBX1 or HuR decreases the mRNA levels of urea cycle enzymes induced by TET2 deficiency. *n* = 3 biologically independent samples per group. **d** Knockdown of YBX1 or HuR reduces the protein levels of urea cycles enzymes and activation of mTORC1 induced by TET2 deficiency. **e** TET2 destabilizes mRNA by abolishing the function of YBX1 and HuR. **f** Overlay of mRNAs upregulated in *sgTET2* group and downregulated in both *shYBX1* and *shHuR* groups in RNA-seq analyses. *n* = 3 biologically independent samples per group. **g** Top motifs in 3′ UTR of overlapped mRNAs upregulated in *sgTET2* group and downregulated in both *shYBX1* and *shHuR* groups. **h**, **i** The combination of TET2, YBX1 and ASL/ASS1 expression is a potential marker for prognosis of LIHC. Survival analyses of 349 LIHC samples from TCGA database stratified by expression levels of TET2, YBX1, ASL (**h**), or TET2, YBX1, ASS1 (**i**).

Next, we tried to unravel how TET2 destabilizes mRNA of urea cycle enzymes through oxidation. YBX1 is a specific 5mC “reader” of mRNAs and stabilizes its target mRNAs by recruiting HuR^38^, which made us hypothesize that TET2 functions as a 5mC “eraser” of mRNA by oxidation to promote decay of its target mRNAs. To verify this hypothesis, we first examined whether TET2-mediated oxidation of 5mC to 5hmC in *ASL* mRNA affects YBX1 and HuR binding and found that binding of these two proteins to mRNA with 5hmC rather than 5mC is dramatically decreased (Fig. 5a). Furthermore, YBX1 and HuR bind to mRNAs of urea cycle enzymes (Fig. 5b), but not *GAPDH*, *TNS4* and *ALB* in vivo (Supplementary Fig. S5i), as determined by qPCR of these two proteins’ RIP products. Importantly, this binding can be enhanced by deletion of TET2 (Fig. 5b). Next, we checked whether YBX1 and HuR affect the expression levels of urea cycle enzymes and mTORC1 signaling. Results showed that YBX1 or HuR knockdown decreases the expression of urea cycle enzymes, and rescues the upregulation of these genes (Fig. 5c) and activation of mTORC1 signaling (Fig. 5d) induced by knockout of *TET2*, while overexpression of YBX1 or HuR exerts minor effects on mTORC1 activity (Supplementary Fig. S5j), indicating TET2-mediated oxidation of mRNA is required for this regulation. Notably, knockdown of YBX1 or HuR fails to rescue the mRNA expression changes of *GAPDH*, *TNS4* and *ALB* caused by deletion of TET2 (Supplementary Fig. S5k), underscoring that TET2-mediated regulation of mRNA stability is more likely gene-specific. Collectively, these results demonstrate that TET2 destabilizes mRNA of urea cycle enzymes by abolishing the function of YBX1 and HuR through mRNA oxidation, and suppresses mTORC1 signaling via YBX1/HuR-urea cycle axis (Fig. 5e). To determine the importance of TET2-YBX1-HuR axis in the regulation of mRNA stability genome wide, we performed RNA-seq analysis of *sgTET2*, *shYBX1* and *shHuR* cells to obtain genome-wide data. As shown in Fig. 5f, 1352 genes are overlapped in three groups (Fig. 5f), that is, genes upregulated in *sgTET2* group and downregulated in both *shYBX1* and *shHuR* groups, suggesting that YBX1-HuR axis is responsible for ~24.6% of upregulated genes in *sgTET2* cells and TET2-YBX1-HuR axis may be involved in the regulation for mRNA stability of various genes. Furthermore, by analyzing the specificity of the regulation by TET2-YBX1-HuR axis, several motifs are identified in the 3′ UTRs of those 1352 genes, most of which contain multiple cytosines (Fig. 5g). Taken together, these results suggest that the modulation of TET2-YBX1-HuR axis on mRNA stability is not restricted to mRNAs of urea cycle enzymes but may represent a specific regulation to the genes with these motifs. As TET2-YBX1-urea cycle axis suppresses mTORC1 signaling, we wondered whether the expression levels of these genes are associated with prognosis of patients with liver cancer. We classified patients into three groups according to their TET2, YBX1 and ASL or ASS1 expression levels: (1) high expression of TET2, low expression of YBX1 and ASL or ASS1 (TET2^high^/YBX1^low^/ASL^low^ or ASS1^low^); (2) low expression of TET2, high expression of YBX1 and ASL or ASS1 (TET2^low^/YBX1^high^/ASL^high^ or ASS1^high^); (3) others. Further analysis identified prominent differences in overall survival among these three groups, with the best outcome for patients in the TET2^high^/YBX1^low^/ASL^low^ or ASS1^low^ group and the worst outcome for those in the TET2^low^/YBX1^high^/ASL^high^ or ASS1^high^ group (Fig. 5h, i), demonstrating TET2-YBX1-urea cycle axis is not only vital for the suppression of mTORC1 signaling, but also represents clinical relevance for prognosis of liver cancer.

### TET2 deficiency sensitizes tumor cells to mTORC1 inhibition

**Fig. 6:**
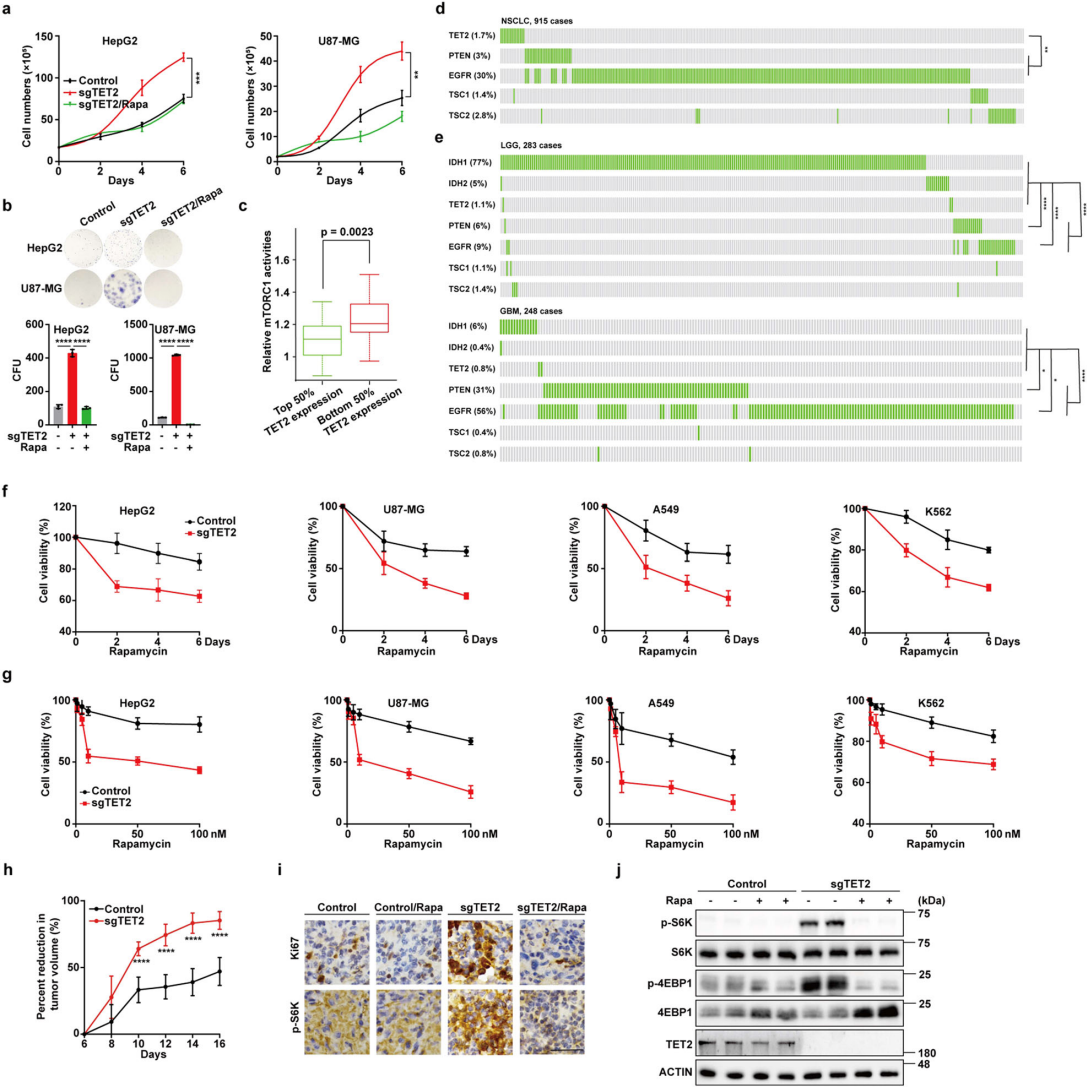
TET2 deficiency sensitizes tumor cells to mTORC1 inhibition. **a** *TET2* KO-induced growth advantage was reversed by rapamycin treatment. Cells were treated with 10 nM rapamycin as indicated. *n* = 3 biologically independent samples per group. **b** *TET2* KO-promoted colony formation was reversed by rapamycin treatment. Cells were treated with 10 nM rapamycin. *n* = 3 biologically independent samples per group. **c** A total of 371 LIHC tumors from TCGA database were divided into two groups based on *TET2* mRNA levels (top and bottom 50% TET2 expression), and their relative mTORC1 activities were qualified and plotted as described in Materials and methods. **d**, **e** TET2 and EGFR mutations, as well as PTEN-EGFR mutations, are mutually exclusive, respectively, in NSCLC (**d**). Mutations in IDH1/2-TET2 axis and mTORC1 signaling (including PTEN and EGFR mutations alone, respectively, and PTEN-EGFR mutations) display mutual exclusivity in LGG and GBM (**e**). Each vertical lane represents one patient, while green lines indicate the presence of mutations. Statistical analysis was performed using the Fisher’s exact test. **f**, **g** TET2 deficiency sensitizes tumor cells to rapamycin treatment in vitro. WT and *TET2* KO tumor cells were treated with 100 nM rapamycin for varying days as indicated (**f**), or different concentrations of rapamycin as indicated for 6 days (**g**). *n* = 4 biologically independent samples per group. **h** TET2 deficiency sensitizes tumor cells to rapamycin treatment in vivo. WT and *TET2* KO HepG2 cells were subcutaneously injected into the left flanks of athymic nude mice. Tumor inhibition rate of rapamycin was measured and calculated every other day as indicated. *n* = 6 biologically independent animals per group. **i** IHC analyses of tumors from nude mice were performed with indicated antibodies. Scale bar, 20 μm. *n* = 3 biologically independent animals per group. **j** Western blot analyses of tumors from nude mice were performed with indicated antibodies. *n* = 2 biologically independent animals per group.

Lastly, we determined the functional significance of the TET2-mTORC1 axis in tumor growth and found that *TET2* KO significantly strengthens capacity for cell proliferation (Supplementary Fig. S6a) and colony formation (Supplementary Fig. S6b) of tumor cells, as well as proliferation of mouse primary liver cells (Supplementary Fig. S6c). Consistent with previous results, TET2-mediated inhibition of cell proliferation is largely dependent on its activity (Supplementary Fig. S6d). More importantly, the effect of *TET2* KO on cell proliferation can be reversed by knockdown of ASL (Supplementary Fig. S6e) or rapamycin treatment (Fig. 6a, b), while arginine supplementation fails to rescue the viability of *TET2* KO cells under rapamycin treatment (Supplementary Fig. S6e). Besides, overexpression of ASL or ASS1 promotes cell proliferation (Supplementary Fig. S6f), indicating that TET2-urea cycle-mTORC1 axis may play an important role in tumor growth. To confirm this hypothesis, we conducted *WDR24*, a component of GATOR2, and *TET2* single/double KO in liver cancer cells and examined their effects on mTORC1 activity and tumor growth using xenograft model. *WDR24* KO abolishes mTORC1 activation (Supplementary Fig. S6g) and tumor growth advantage induced by TET2 deficiency (Supplementary Fig. S6h, i). As TET2 deficiency strongly activates mTORC1 and promotes tumor growth, we wondered whether *Tet2* KO also affects growth of mice. We monitored the weight of WT and *Tet2* KO mice for several weeks and found that *Tet2* KO displays a minor effect on mouse weight (Supplementary Fig. S6j). To provide clinical evidence for TET2-mediated mTORC1 suppression, we analyzed 371 liver hepatocellular carcinoma (LIHC) tumor samples from TCGA database. An inverse correlation between TET2 expression and mTORC1 activity is recapitulated in LIHC tumors. Patients with lower TET2 expression level exhibit higher mTORC1 activity (Fig. 6c). PTEN and EGFR are upstream regulators of mTORC1 signaling pathway^39^ and are frequently mutated in many cancers. TCGA database analysis demonstrated that TET2 and EGFR mutations, as well as PTEN-EGFR mutations, are mutually exclusive, respectively, in non-small cell lung cancer (NSCLC) (Fig. 6d). IDH1/2 mutations are frequent in low-grade gliomas (LGG) and glioblastomas (GBM), which produce oncometabolite 2-HG and inhibit TET2 activity. Further analysis showed that mutations in IDH1/2-TET2 axis and mTORC1 signaling (including PTEN and EGFR mutations alone, respectively, and PTEN-EGFR mutations) display mutual exclusivity in LGG and GBM (Fig. 6e), suggesting that abrogation of PTEN function or oncogenic activation of EGFR might not confer a growth advantage to tumors carrying IDH1/2 and TET2 mutations, or that IDH1/2 and TET2 mutations might mimic PTEN loss or EGFR mutation-induced mTORC1 activation, which leads us to hypothesize that tumors with loss-of-function mutations in TET2 might be sensitive to mTORC1 suppression. Fortunately, *TET2* KO significantly sensitizes a variety of tumor cells to mTORC1 inhibition by rapamycin (Fig. 6f, g), temsirolimus (Supplementary Fig. S6k, l) or everolimus (Supplementary Fig. S6m, n), which is recaptured in mouse model. Rapamycin treatment dramatically diminishes the *TET2* KO tumor growth, while only has moderate efficacy to WT tumors (Fig. 6h and Supplementary Fig. S6o). These observations are consistent with the IHC staining results of Ki67 and p-S6K (Fig. 6i), and western blot against p-S6K and p-4EBP1 (Fig. 6j) of tumors from all four groups. These results demonstrate that various tumors with TET2 mutations or inactivation are more sensitive to mTORC1 inhibition.

## Discussion

In summary, this study establishes a functional link between TET2 and mTORC1, two major regulators of tumor growth, and provides a molecular mechanism through which TET2 suppresses mTORC1 signaling. TET2 acts as a 5mC “eraser” of mRNA, which disrupts YBX1–HuR binding and destabilizes mRNA of urea cycle enzymes, thus negatively regulating urea cycle, which is the main source of cellular arginine biosynthesis. Collectively, TET2 suppresses mTORC1 signaling through decreasing arginine level in vivo, thereby inhibiting cell growth and activating autophagy (Supplementary Fig. S7). In addition, TET2 represses tumor cell growth and colony formation by inactivating mTORC1 signaling, providing a new mechanism for TET2-mediated suppression of tumor growth. TET2 activity is inhibited as tumor growth proceeds, leading to urea cycle activation and arginine production, which in turn activates mTORC1 signaling to enable cell growth, and then proliferation, resulting in the eventual tumor growth.

Recently, the roles of TET2 in atherosclerosis development^40^, inflammation resolution^41^ and pathogen infection-induced myelopoiesis^20^ independently of DNA methylation have been gradually uncovered. Our study provides further evidence for DNA dioxygenase-independent function of TET2. C-terminal of TET2 is responsible for its binding with RNAs^42^, while TET1 and TET3 are lack of this RNA-binding region. This region may be crucial for binding of TET2 and RNAs. The specificity of TET2-mediated mRNA stability regulation is interesting and worthy of further investigation.

TET2 acts as a tumor suppressor, which is mutated or inactivated in various types of tumors, while the physiological roles of TET2 in normal cells still remain elusive. Given the vital roles of TET2-mTORC1 axis, it would be of great interest and of importance to explore the interplay of these genes in normal cells. The determination of organ size is achieved by coordinating of diverse cellular signaling pathways involved in regulation of cell number and size^43^. mTORC1 functions as a pivotal nutrient sensor in cells and is involved in regulation of many physiological processes, including lipid, nucleotide and glucose metabolism, autophagy and protein synthesis, to regulate cell growth^27,44^. Besides, as a downstream effector of various frequently mutated pathways such PI3K signaling, imbalance of mTORC1 activity can lead to metabolic dysregulation and diseases^45^. Therefore, TET2 is supposed to be capable of maintaining cell size and metabolic homeostasis and providing protection against tumorigenesis and metabolic disorders by suppressing mTORC1 activity in normal cells.

Our study not only uncovers a novel function of TET2 in suppressing mTORC1 signaling and inhibiting cell growth, but also links epigenetic regulation to cell metabolism via mRNA oxidation. Furthermore, the link between TET2 and urea cycle/mTORC1 and the feedback regulation of TET2 by mTORC1 signaling support an integration of epigenetic regulation, metabolism, and nutrient status in the regulation of cell growth control. The functional crosstalk between TET2 and mTORC1 revealed in this study not only has important implications in cancer biology, but also provides therapeutic strategy for the clinical treatment of various tumors with TET2 mutations or inactivation.

## Materials and methods

### Cell culture and plasmids transfection

Cells were cultured in DMEM (Meilun Biotechnology, China) containing 1× antibiotics (containing Penicillin-Streptomycin-Amphotericin B) and 10% FBS (Biological Industries, Israel) and saved with CELLSAVING (New Cell & Molecular Biotech). Glutamine-deprived and arginine-deprived DMEM was obtained from NJJCBIO (China) and Meilun Biotechnology (China). Plasmids were transfected into cells with EZ Trans (Life-iLab, China) and X-tremeGENE HP DNA transfection reagent (Roche) following the manuals. Rapamycin, temsirolimus and everolimus for cell viability assay were purchased from Meilun Biotechnology, China.

### RNA extraction, qPCR and RNA-seq

The total RNA was extracted using EZ-press RNA purification kit (EZ Bioscience, USA). Total RNA was reverse-transcribed by EZ Bioscience-RT mix (EZ Bioscience, USA). qPCR was performed using Hieff^®^ qPCR SYBR Green Master Mix (No Rox) (Yeasen, China). Primers used in this study are shown in Supplementary Tables S1–S8. All primers were synthesized by BioSune (Shanghai, China). RNA-seq analysis was conducted by Origin-gene (Shanghai, China).

### Whole-genome bisulfite sequencing

Whole-genome bisulfite sequencing and data analysis were conducted by Acegen (Shenzhen, China).

### Western blot

For western blot sample preparation, cells or tissues were washed in PBS for three times, followed by treatment with 0.5% NP-40 lysis buffer containing 1× protease inhibitor cocktail (APExBIO, USA). For phosphorylation sample preparation, 1× phosphatase inhibitor cocktail (TargetMol, China) was added into the lysate. EZ Protein any KD PAGE kit (Life-iLab, China) was applied for SDS-PAGE electrophoresis. For liver tissue phosphorylation sample, 4%–12% Precast Bis-Tris Gel (Tanon, China) was used. The images were taken using Tanon 5200 imaging system (Tanon, China).

### Antibodies

The antibodies used in this study are listed below: 4EBP1 (CST, 9644T), p-4EBP1 (Thr37/46) (CST, 2855T), p-S6 (Ser235/236) (CST, 4858T), S6K (CST, 2708T), p-S6K (Thr389) (CST, 9234T), ULK1 (CST, 8054T), p-ULK1 (Ser757) (CST, 14202T), TET2 (CST, 18950S), ACTIN (Proteintech, 60008-1-Ig), ASL (Proteintech, 16645-1-AP), HuR (Proteintech, 11910-1-AP), WDR24 (Proteintech, 20778-1-AP), YBX1 (Proteintech, 20339-1-AP), AKT (HUABIO, ET1609-47), p-AKT (Ser473) (HUABIO, ET1607-73), ARG1 (HUABIO, ET1605-8), ASS1 (HUABIO, R1608-5), CPS1 (HUABIO, ET7107-69), Ki67 (HUABIO, ET1609-34), LC3 (HUABIO, ET1701-65), OTC (HUABIO, ER1914-49), S6 (HUABIO, ER64863), PKCα (HUABIO, ET1608-15), p-PKCα (Thr638) (HUABIO, ET1702-17), SGK1 (HUABIO, ET1610-19), TET1 (Absci, AB38243), TET3 (SAB, 30014), p-SGK1 (Ser422) (SAB, 12220), CASTOR1 (Abclonal, A20710), SLC38A9 (Novus, NBP1-69235).

### IHC analysis

IHC analysis was performed as described previously^46^.

### Flow cytometry

Cells were fixed with 75% ethanol/PBS at 4 °C overnight and stained for 30 min following the instruction of cell cycle kit (Meilun Biotechnology, China). Cell cycle was analyzed by BD LSR II flow cytometer and FlowJo vX.0.7 software.

### siRNA and CRISPR-Cas9 sgRNA preparation

*siARG1*, *siASS1*, *siASL*, *siHuR* and *siYBX1* were purchased from RiboBio (Guangzhou, China). 100 nM siRNAs were transfected into cells with Lipofectamine 2000 (Thermo, USA). sgRNAs of *TET1*, *TET2*, *TET3* and *WDR24* were cloned into pLentiCRIPSR v2 vector. CRISPR-Cas9 lentivirus was produced by transfecting 6 μg sgRNA plasmid, 4.5 μg psPAX2 and 1.5 μg pMD2.G into HEK-293T cells in 100-mm dishes. Supernatant was collected at 48 h and 72 h after transfection and stored at –80 °C for infection. The sequences of siRNAs and sgRNAs are listed as below:

*siARG1*#1 (5′-GGACUGGACCCAUCUUUCA-3′),

*siARG1*#2 (5′-GAAGUAACUCGAACAGUGA-3′),

*siASS1*#1 (5′-GGAAUGAAGUCCCGAGGUA-3′),

*siASS1*#2 (5′-GGAGCAAGGCUAUGACGUC-3′),

*siASL* (5′-GCAUGGAUGCCACUAGUGA-3′),

*siHuR* (5′-AAGAGGCAAUUACCAGUUUCA-3′),

*siYBX1* (5′-GCAGACCGUAACCAUUAUATT-3′),

*sgTET1* (5′-ACAAAGTTCATGCAACACGG-3′),

*sgTET2*#1 (5′-GATTCCGCTTGGTGAAAACG-3′),

*sgTET2*#2 (5′-TACCGTTCAGAGCTGCCACC-3′),

*sgTET3* (5′-GAAAGCCATCCGGATCGAGA-3′),

*sgWDR24* (5′-CACGAACTGTTCCTCCTCGA-3′),

*shYBX1* (5′-GAGAACCCTAAACCACAAGAT-3′),

*shHuR* (5′-GCAGCATTGGTGAAGTTGAAT-3′).

### Animal model

*Tet2* KO mice were purchased from The Jackson Laboratory (USA). For transplantation of HepG2 cells or MHCC97H cells to BALB/c nude mice, cells were concentrated to 10^6^ cells per 100 μL PBS, which were then mixed with equal volumes of Matrigel (Corning). Overall, 200 μL cell mixtures were subcutaneously injected into the flank of 6-week-old male BALB/c nude mice. For rapamycin treatment, rapamycin in ethanol at 10 mg/mL was diluted in 5% Tween-80 and 5% PEG-400 (Meilun Biotechnology, China). Treatment was conducted by intraperitoneal injection of 3 mg/kg every other day starting at day 4 (the day of transplantation). Tumor volume was calculated as volume = width^2^ × length × 0.5. All animal experiments were approved by the ethic committee of School of Basic Medical Sciences, Fudan University. All animals used in this study received appropriate care according to institutional guidelines.

### Measurement of metabolites

Cells were seeded in 100-mm dishes at 80% confluency and cultured overnight. Total metabolites were collected with 80% cold methanol after thawing in liquid nitrogen for three times. For tissue metabolite extraction, liver tissue was directly homogenized in 80% cold methanol. Supernatant containing total metabolites was collected for liquid chromatography-mass spectrometry (LC-MS) analysis. LC was performed with solvent A (H_2_O) and solvent B (ACN) through Aq-C18 column (Shimadzu). The protocol is: 5% solvent B for 1 min, 90% solvent B for 20 min, 5% solvent B for 12.1 min. MS detection was performed on SCIEX 4000 Q TRAP.

### Quantification of ammonia, inhibition rate and urea release

Cellular ammonia and urea release were analyzed by using an ammonia and urea quantification kit from NJJCBIO (China). Inhibition rate of ammonia was measured with CCK-8 kit (Meilun Biotechnology, China).

### TET2/YBX1/HuR RIP assay

HepG2 cells were seeded at 80%–90% confluency in 100-mm dishes prior to experiment. 1% formaldehyde was used to fix cells at room temperature for 10 min, followed by treatment with glycine at the final concentration of 125 mM to quench unreacted formaldehyde. Cells were scraped into a separate 1.5-mL microcentrifuge tube and the pellet cells were collected for sonication in 500 μL lysis buffer (1% SDS, 10 mM EDTA, 50 mM Tris-HCl, pH 8.1) with protease inhibitor cocktail and RNase inhibitor after washing the cells with cold PBS for three times. After sonication, supernatant was collected for RIP assay. 50 μL supernatant was mixed with 450 μL dilution buffer (16.7 mM Tris-HCl, 0.01% SDS, 1.1% Triton X-100, 1.2 mM EDTA, pH 8.1, 167 mM NaCl) containing protease inhibitor cocktail. TET2, YBX1 or HuR antibody or IgG and 15 μL fully resuspended protein A/G magnetic beads (Millipore) were added to the mixture and rotated at 4 °C overnight. After incubation, the beads were collected and washed with low salt wash buffer (150 mM NaCl, 20 mM Tris-HCl, pH 8.1, 1% Triton X-100, 0.1% SDS, 2 mM EDTA), high salt wash buffer (1% Triton X-100, 20 mM Tris-HCl, pH 8.1, 0.1% SDS, 500 mM NaCl, 2 mM EDTA) and LiCl wash buffer (1% sodium deoxycholic acid, 10 mM Tris-HCl, pH 8.1, 1% NP40, 0.25 M LiCl, 1 mM EDTA). At last, 500 μL elution buffer (100 mM NaHCO_3_, 1%SDS) with Proteinase K was added to beads and incubated at 60 °C for 2 h to elute RNA. The RNA was purified with TRIzol (Thermo) according to the manufacturer’s instructions. For qPCR quantification of RIP assay, 200 ng RNA was used for cDNA preparation.

### 5mC/5hmC RIP assay

RNA for 5mC/5hmC RIP assay was extracted using EZ-press RNA purification kit (EZ Bioscience, USA). In total, 10 μg RNA in 300 μL RNase-free water was fragmented by sonication. For 5mC RIP, 50 μL fragmented RNA was mixed with 10 μL Buffer C (Active motif), 2 μL 5mC antibody (Active motif) or IgG, 2 μL Bridging antibody (Active motif) and protease inhibitor. For 5hmC RIP, 50 μL fragmented RNA was mixed with 10 μL Buffer C (Active motif), 4 μL 5hmC antibody (Active motif) or IgG, 2 μL Bridging antibody (Active motif) and protease inhibitor. 50 μL fully resuspended protein A (Shanghai Genomics, China) beads were added to each mixture and rotated at 4 °C overnight. After incubation, beads were collected and washed with cold Buffer C and Buffer D (Active motif). Beads were resuspended with 50 μL Elution Buffer AM2 (Active motif) and incubated at 4 °C for 15 min. 50 μL Neutralization Buffer (Active motif) was added to the mixture and RNA was ready to use. The RNA was further purified with TRIzol (Thermo). For qPCR quantification of RIP assay, 200 ng RNA was used for cDNA preparation.

### In vitro mRNA oxidation assay

Reaction mixture contains *ASL* mRNA with 5mC modification, TET2 CD, oxidation reagent 1 and oxidation reagent 2. *ASL* mRNA with 5mC modification was synthesized by BioSune (China) and the sequence is GCAAGGUG(5mC)GAGGAUGCUUG. TET2 CD was purified from HEK-293T cells after overexpression of exogenous TET2 CD. Oxidation reagent 1 is 1.5 mM Fe(NH_4_)_2_(SO_4_)_2_·6H_2_O. Oxidation reagent 2 contains 333 mM NaCl, 167 mM HEPES (pH 8.0), 4 mM ATP, 8.3 mM DTT, 3.3 mM α-KG and 6.7 mM l-ascorbic acid. The final reaction volume is 25 μL containing 250 ng ASL mRNA, 2 μL oxidation reagent 1, 8 μL oxidation reagent 2 and 3 μg TET2 CD with H_2_O to make up to 25 μL. The mixture was incubated at 37 °C for 2 h, followed by purification with spin column (Sangon Biotech, China). The mixture was digested with S1 nuclease (Takara) and Alkaline phosphatase (Takara) to prepare single nucleotides for LC-MS analysis of 5mC and 5hmC levels of the products. Both 5mC and 5hmC-modified *ASL* mRNA fragments were validated with LC-MS before pull-down analysis.

### Biotin-RNA 5mC/5hmC pull-down assay

Pull down assay was conducted by incubating the lysate of HepG2 cells with biotin-*ASL* mRNA 5mC (GCAAGGUG(5mC)GAGGAUGCUUG) and 5hmC (GCAAGGUG(5hmC)GAGGAUGCUUG). Complexes were precipitated with streptavidin beads (Beyotime, China), followed by immunoblot analysis with indicated antibodies.

### TCGA RNA-seq analysis

Correlation analysis of expression levels of TET2 and urea cycle enzymes was performed through GEPIA database (http://gepia.cancer-pku.cn/). Raw RNA-seq data for 371 LIHC tumor tissues were downloaded from TCGA database. Raw data of each sequenced gene were rescaled to set the median equal to 1. The tumor tissues were divided into two groups according to the median value of TET2. mTORC1 activities were quantified by averaging the normalized expression of 30 mTORC1 target genes involved in glycolysis, pentose phosphate pathway, fatty acid biogenesis, oxidative phosphorylation, ribosome and lysosome biogenesis, that is, *PFKL*, *TPI1*, *PGM1*, *PGD*, *TALDO1*, *ACSS2*, *MVK*, *SC5D*, *HSD17B7*, *GGPS1*, *HSD17B12*, *FADS2*, *AGPAT5*, *COX5A*, *CYCS*, *NDUFS8*, *NOP14*, *NOP56*, *RRP9*, *RRP12*, *ARSB*, *ATP6V1H*, *CLCN7*, *CTSB*, *GALNS*, *GNS*, *LAMP1*, *PSAP*, *SGSH* and *TPP1*. For mutual exclusivity analysis, RNA-seq data of NSCLC, LGG and GBM were downloaded from http://www.cbioportal.org. Statistical analysis was performed using the Fisher’s exact test.

### Statistics and reproducibility

All quantitative data were presented as the means ± standard deviation (SD) and analyzed by GraphPad Prism 8 software using two-sided unpaired Student’s *t*-test. *P < 0.05, **P < 0.01, ***P < 0.001.

### Supplementary information

Supplementary information

## Data Availability

RNA-seq and whole-genome bisulfite sequencing data in this project are available at the NCBI SRA database under accession numbers PRJNA781771 and PRJNA782072, respectively. RNA-seq data for overlapped analyses of mRNAs in *sgTET2*, *shYBX1* and *shHuR* are available at the NCBI SRA database under accession number PRJNA874356. All other data supporting the findings of this study are available from the corresponding author upon request.
