# Publisher Correction: TET2 is required to suppress mTORC1 signaling through urea cycle with therapeutic potential

**DOI:** 10.1038/s41421-023-00600-9

**Published:** 2023-09-21

**Authors:** Jing He, Mingen Lin, Xinchao Zhang, Ruonan Zhang, Tongguan Tian, Yuefan Zhou, Wenjing Dong, Yajing Yang, Xue Sun, Yue Dai, Yue Xu, Zhenru Zhang, Ming Xu, Qun-Ying Lei, Yanping Xu, Lei Lv

**Affiliations:** 1 MOE Key Laboratory of Metabolism and Molecular Medicine, Department of Biochemistry and Molecular Biology, School of Basic Medical Sciences, Fudan University, Shanghai, China; 2 Tongji Hospital, Shanghai Key Laboratory of Signaling and Disease Research, Frontier Science Center for Stem Cell Research, School of Life Sciences and Technology, Tongji University, Shanghai, China; 3 Shanghai Key Laboratory of Maternal Fetal Medicine, Clinical and Translational Research Center of Shanghai First Maternity and Infant Hospital, Tongji University, Shanghai, China; 4 UConn Center on Aging, UConn Health, Farmington, CT USA; 5 Fudan University Shanghai Cancer Center and Cancer Metabolism Laboratory, Institutes of Biomedical Sciences, Shanghai Medical College, Fudan University, Shanghai, China

**Keywords:** Cancer metabolism, RNA decay

Correction to: *Cell Discovery* (2023) 9:84

10.1038/s41421-023-00567-7 published online 08 August 2023

After the publication of this article, it was noticed that the ethical document was published as a Supplementary file by mistake. The ethical document has been removed now. We apologize for this error.

