# Supplementary material for: TET2 is required to suppress mTORC1 signaling through urea cycle with therapeutic potential
Source: Cell Discov. 2023 Aug 8;9:84. doi: 10.1038/s41421-023-00567-7 (PMC10406918; doi:10.1038/s41421-023-00567-7)
Supplement: Supplementary file 1 — Supplementary information [file 41421_2023_567_MOESM1_ESM.pdf]

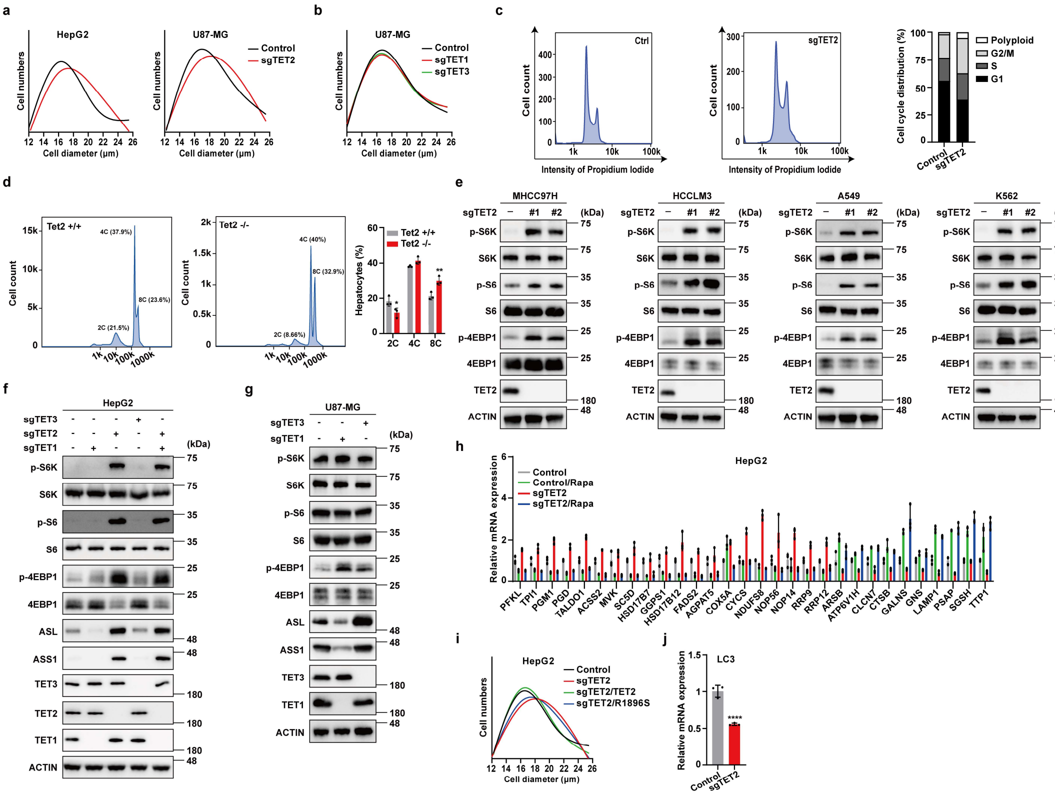

Supplementary Fig. S1 Deficiency of Tet2 activates mTORC1 signaling. (**a**) Depletion of TET2 increases cell size of tumor cells. (**b**) Depletion of TET1 or TET3 fails to increase cell size of U87-MG. (**c**) TET2 depletion increases the percentage of G2/M and polyploidy phase of cells. Cell cycle distribution was determined by propidium iodide staining. n = 3 biologically independent samples per group. (**d**) Percentage of polyploidy phase is increased in primary hepatocytes of Tet2-/- mice. 2C, 4C and 8C DNA content corresponding to diploid, tetraploid and octaploid hepatocytes, respectively. n = 3 biologically independent animals per group. (**e**) Depletion of TET2 increases phosphorylation level of S6K, S6 and 4EBP1 in tumor cells as indicated. (**f, g**) Depletion of TET1 or TET3 fails to activate mTORC1 signaling in HepG2 (f) and U87-MG (g). (**h**) TET2 mediated regulation of mTORC1 target genes can be rescued by rapamycin. 30 mTORC1 target genes involved in several metabolism pathways were employed to verify the TET2-mTORC1 axis in tumor cells. n = 3 biologically independent samples per group. (**i**) Catalytic mutant TET2 (R1896S) has no effect on cell size. (**j**) LC3 mRNA level is decreased in sgTET2 HepG2. n = 3 biologically independent samples per group.

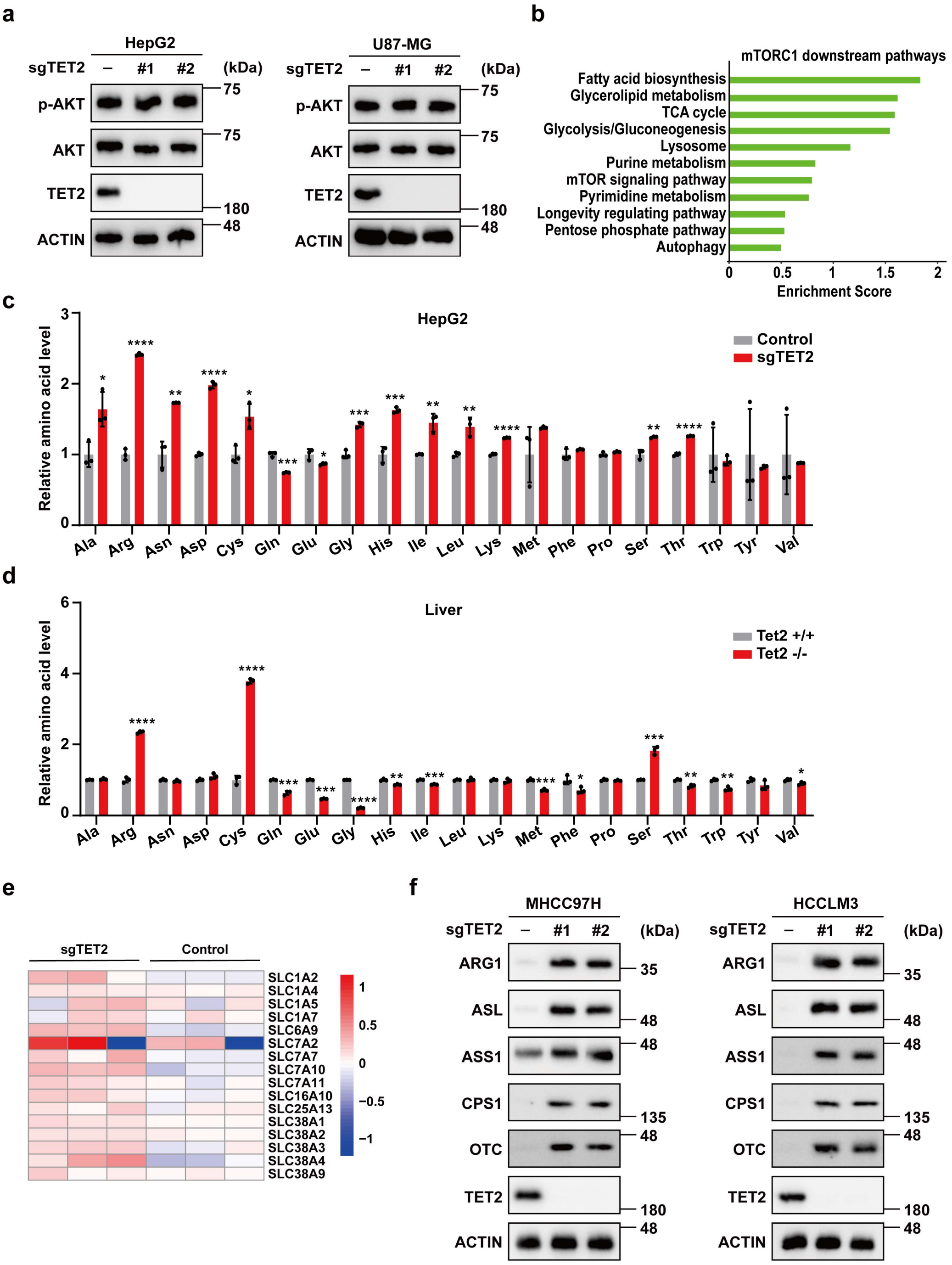

Supplementary Fig. S2 TET2 is a negative regulator of urea cycle. (**a**) Depletion of TET2 has no effect on phosphorylation of AKT. (**b**) KEGG analysis of mTORC1 downstream pathways in sgTET2 HepG2 cells. n = 3 biologically independent samples per group. (**c, d**) Depletion of TET2 regulates several amino acids in tumor cell (c) and livers (d), of which arginine is one of significant changed amino acids in both groups.

n = 3 biologically independent samples per group. (**e**) Heatmap of amino acids transporters in RNA-seq data. (**f**) TET2 knockout increases protein levels of urea cycle enzymes in tumor cells as indicated.

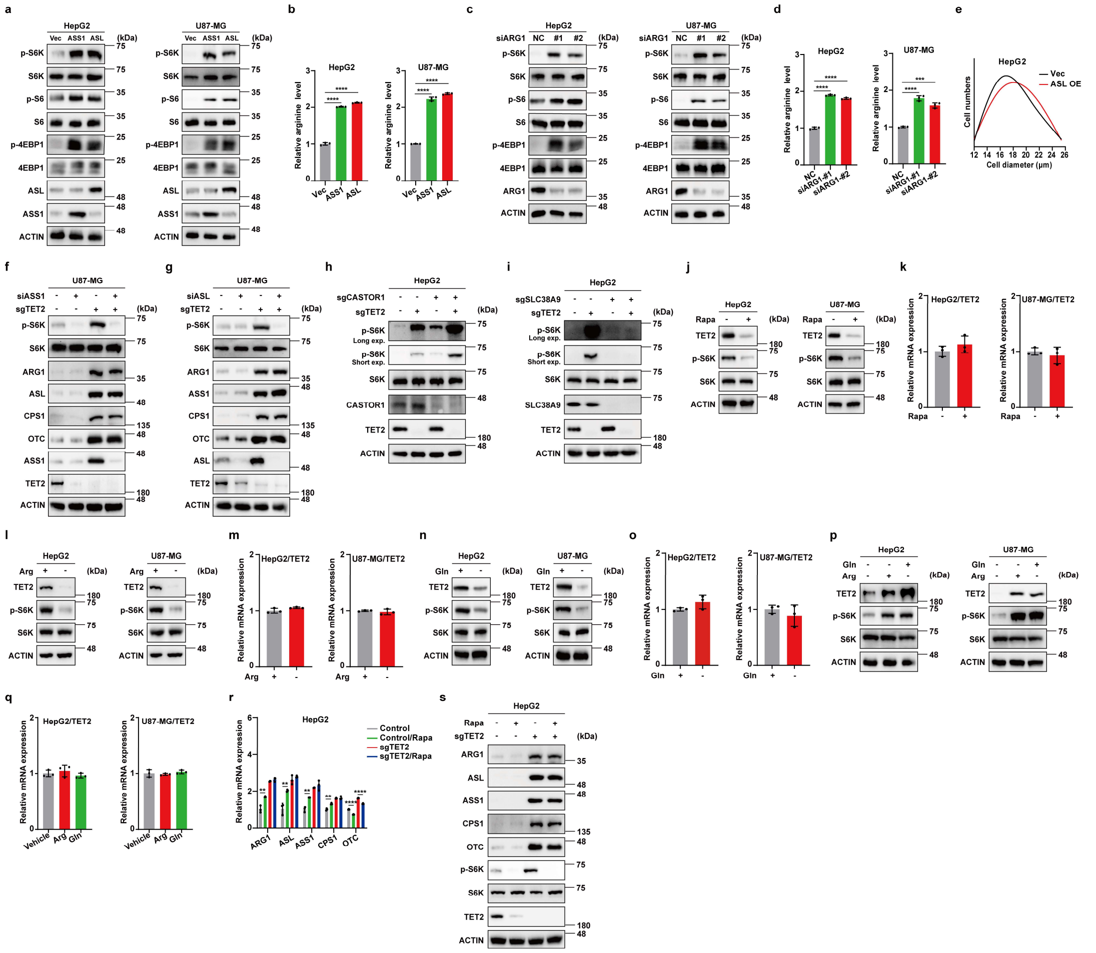

Supplementary Fig. S3 TET2 suppresses mTORC1 activation via urea cycle. (a) Overexpression of arginine-producing enzymes ASL or ASS1 enhances mTORC1 signaling. (**b**) Overexpression of arginine-producing enzymes ASL or ASS1 enhances cellular arginine level. n = 3 biologically independent samples per group. (**c**) Knockdown of arginine-consuming enzyme ARG1 enhances mTORC1 signaling. (**d**) Knockdown of arginine-consuming enzyme ARG1 enhances cellular arginine level. n = 3 biologically independent samples per group. (**e**) Overexpression of ASL increases cell size of HepG2. (**f, g**) Knockdown of ASS1 (f) or ASL (g) blocks TET2 knockout induced upregulation of mTORC1 activation. (**h**) Knockout of CASTOR1 cannot abolish mTORC1 activation caused by TET2 loss. Long exp, long exposure. Short exp,

short exposure. (**i**) SLC38A9 KO blocks mTORC1 activation caused by TET2 deficiency. Long exp, long exposure. Short exp, short exposure. (**j, k**) Rapamycin treatment reduces TET2 protein level (j), but not mRNA level (k). HepG2 and U87-MG cells were treated with 20 nM rapamycin for 24 h. n = 3 biologically independent samples per group. (**l, m**) Arginine deprivation reduces TET2 protein level (l), but not mRNA level (m). HepG2 and U87-MG cells were deprived of arginine for 24 h. n = 3 biologically independent samples per group. (**n, o**) Glutamine deprivation reduces TET2 protein level (n), but not mRNA level (o). HepG2 and U87-MG cells were deprived of glutamine for 24 h. n = 3 biologically independent samples per group. (**p, q**) Glutamine or arginine stimulation increases TET2 protein level (p), but not mRNA level (q). HepG2 and U87-MG cells were treated with 1 mM glutamine or arginine for 24 h. n = 3 biologically independent samples per group. (**r, s**) Rapamycin treatment exhibits minor or no effect on mRNA (r) and protein (s) level of ARG1, ASL, ASS1 and CPS1 in HepG2, indicating the upregulation of urea cycle enzymes induced by TET2 depletion is the cause of mTORC1 activation. n = 3 biologically independent samples per group.

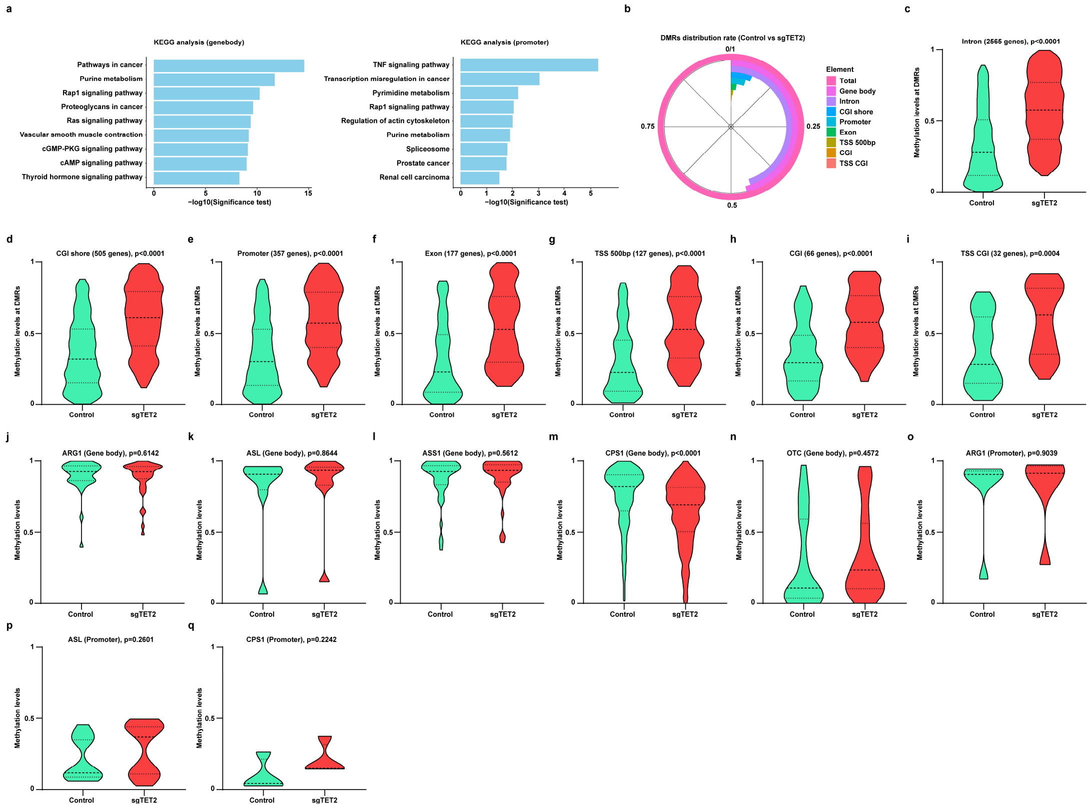

Supplementary Fig. S4 TET2 exerts minor effect on DNA methylation of urea cycle enzymes. (**a**) Methylation levels of numerous genes involved in multiple biological pathways are aberrantly regulated in sgTET2 cells. Whole genome bisulfite sequencing of control and TET2 knockout HepG2 cells and KEGG enrichment analysis were performed. n = 3 biologically independent samples per group. (**b**) Differentially methylated regions (DMRs) of whole genome regulated by TET2 mainly distribute in introns, CGI shores, promoters, exons, transcription start site (TSS) regions and CpG islands (CGIs) of genes. n = 3 biologically independent samples per group. (**c-i**) Methylation levels at DMRs in introns (c), CGI shores (d), promoters (e), exons (f), TSS 500bp (g), CGIs (h) and TSS CGIs (i) of genes are upregulated in sgTET2 cells as indicated. n = 3 biologically independent samples per group. (**j-n**) Methylation levels in gene bodies of ARG1 (j), ASL (k), ASS1 (l) and OTC (n) are not significantly changed, while methylation levels in gene body of CPS1 (m) are decreased in sgTET2 cells as indicated. n = 3 biologically independent samples per group. (**o-q**) Methylation levels in promoters of ARG1 (o), ASL (p) and CPS1 (q) are not significantly changed in sgTET2 cells as indicated. The methylation levels in promoters of ASS1 and OTC

are hard to determine, as there are few CpGs in promoters of ASS1 and OTC. n = 3 biologically independent samples per group.

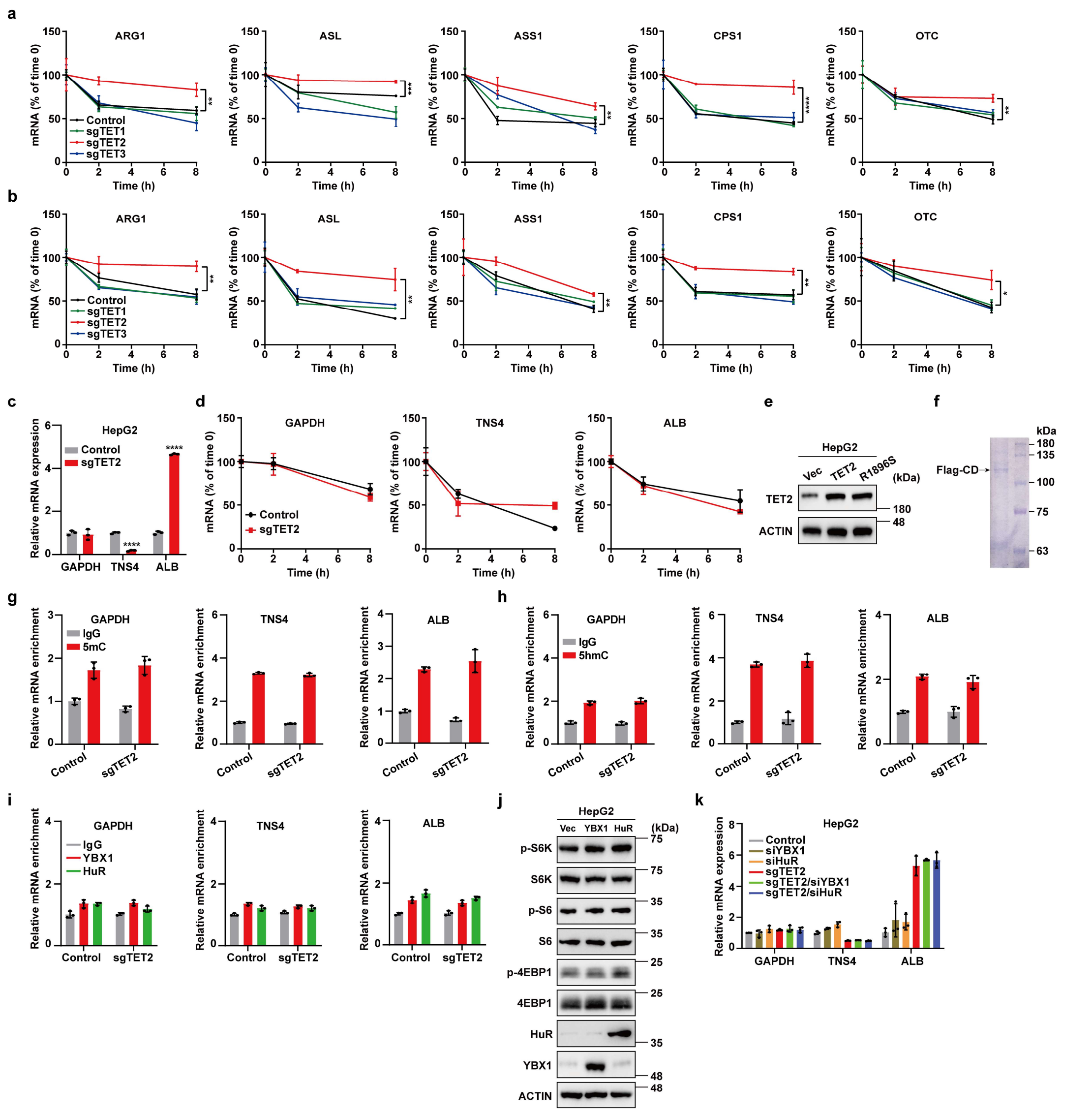

Supplementary Fig. S5 TET2 restrains urea cycle through mRNA oxidation. (**a, b**) Knockout of TET2, but not TET1 or TET3, increases mRNA stability of urea cycle enzymes. HepG2 (a) and U87-MG (b) were treated with 5 μg/ml actinomycin D for different times as indicated. mRNA decay of urea cycle enzymes was quantified by qPCR. n = 3 biologically independent samples per group. (**c**) TNS4 and ALB are differentially expressed in sgTET2 HepG2 while GAPDH remain unchanged. n = 3 biologically independent samples per group. (**d**) Control and TET2 knockout HepG2 were treated with 5 μg/ml actinomycin D for different times as indicated. The mRNA stability of GAPDH, TNS4 and ALB were quantified by qPCR. n = 3 biologically independent samples per group. (**e**) Wild type and catalytic mutant TET2 are overexpressed in HepG2. (**f**) Purified TET2 catalytic domain (TET2-CD) was detected

by coomassie brilliant blue staining. The TET2 CD fragment is indicated with arrow. (**g, h**) TET2 exerts no effect on 5mC (g) and 5hmC (h) levels of GAPDH, TNS4 and ALB mRNAs. mRNAs of GAPDH, TNS4 and ALB were immune-precipitated from control and TET2 knockout HepG2 cells with anti-5mC or 5hmC antibody and quantified by qPCR. n = 3 biologically independent samples per group. (**i**) The binding capacity of YBX1 and HuR to mRNAs of GAPDH, TNS4 and ALB is independent of TET2. n = 3 biologically independent samples per group. (**j**) Overexpression of YBX1 or HuR exerts minor effect on mTORC1 activity. (**k**) Knockdown of YBX1 or HuR cannot rescue the mRNA expression change of GAPDH, TNS4 and ALB caused by deletion of TET2. n = 3 biologically independent samples per group.

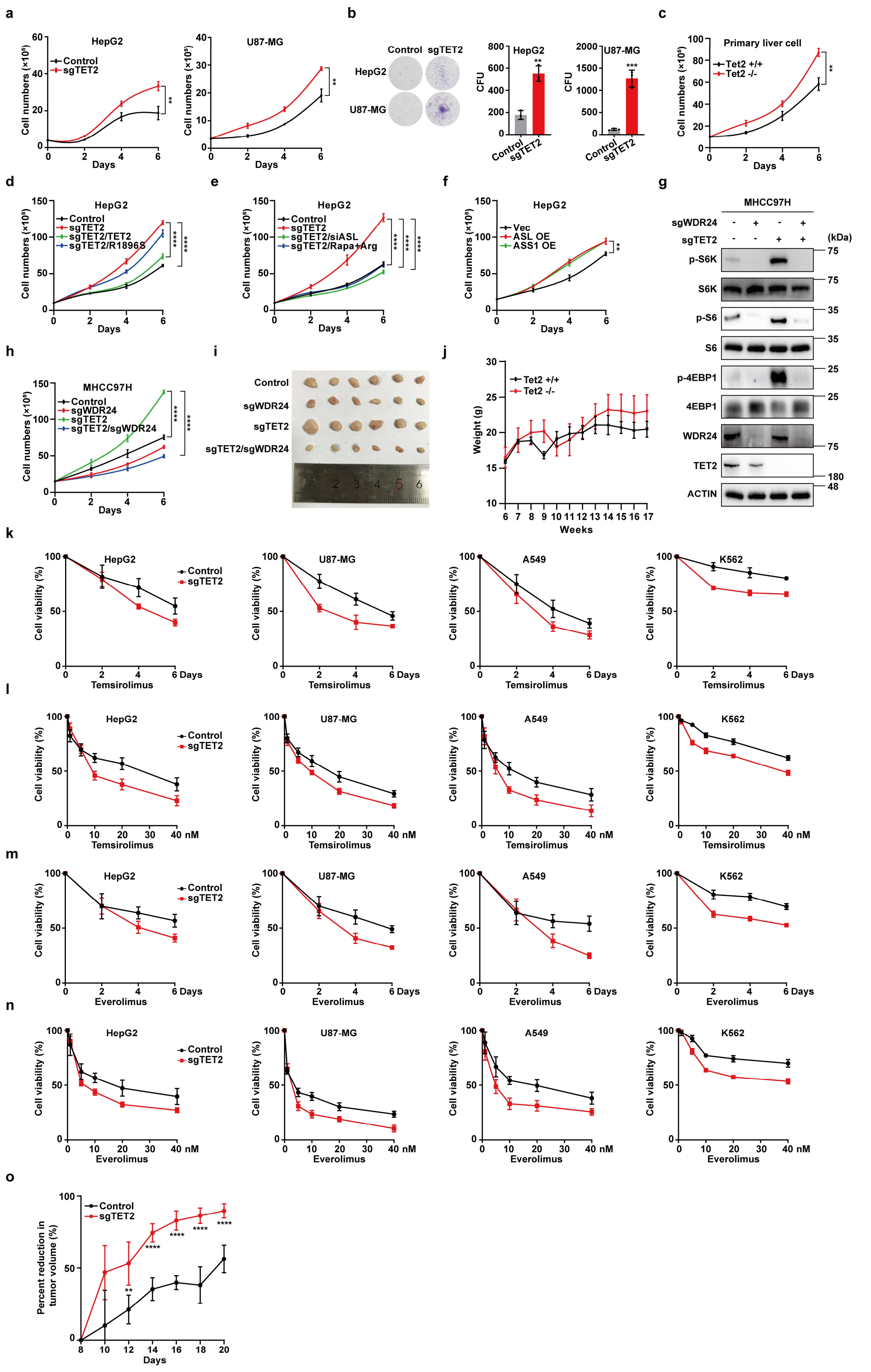

Supplementary Fig. S6 TET2 deficiency sensitizes tumor cells to mTORC1

inhibition. (**a**) TET2 knockout promotes tumor cell proliferation. n = 3 biologically independent samples per group. (**b**) TET2 knockout promotes tumor cell colony formation. n = 3 biologically independent samples per group. (**c**) Tet2 knockout promotes primary liver cell proliferation. n = 3 biologically independent animals per group. (**d**) TET2 mediated inhibition of cell proliferation is dependent on its activity. Catalytic mutant TET2 (R1896S) has no effect on cell proliferation. n = 3 biologically independent samples per group. (**e**) Knockdown of ASL blocks cell proliferation induced by TET2 deficiency. n = 3 biologically independent samples per group. (**f**) Overexpression of ASS1 or ASL promotes cell proliferation. n = 3 biologically independent samples per group. (**g-i**) Knockout of GATOR2 component WDR24 blocks mTORC1 activation (g), cell proliferation (h) and tumor growth (i) induced by TET2 deficiency. n = 3 biologically independent samples per group. (**j**) Tet2 KO displays minor effect on mouse weight. Mouse weight was monitored for 12 weeks. n = 8 biologically independent animals per group. (**k, l**) TET2 deficiency sensitizes tumor cells to temsirolimus treatment *in vitro*. Control and TET2 KO tumor cells were treated with 20 nM temsirolimus for different times as indicated (k), or different concentration of temsirolimus as indicated for 6 days (l). n = 4 biologically independent samples per group. (**m, n**) TET2 deficiency sensitizes tumor cells to everolimus treatment *in vitro*. Control and TET2 KO tumor cells were treated with 20 nM everolimus for different times as indicated (m), or different concentration of everolimus as indicated for 6 days (n). n = 4 biologically independent samples per group. (**o**) TET2 deficiency sensitizes tumor cells to rapamycin treatment *in vivo*. Control and TET2 KO MHCC97H cells were subcutaneously injected into the left flanks of athymic nude mice. Tumor inhibition rate of rapamycin was measured and calculated every other day as indicated. n = 6 biologically independent animals per group.

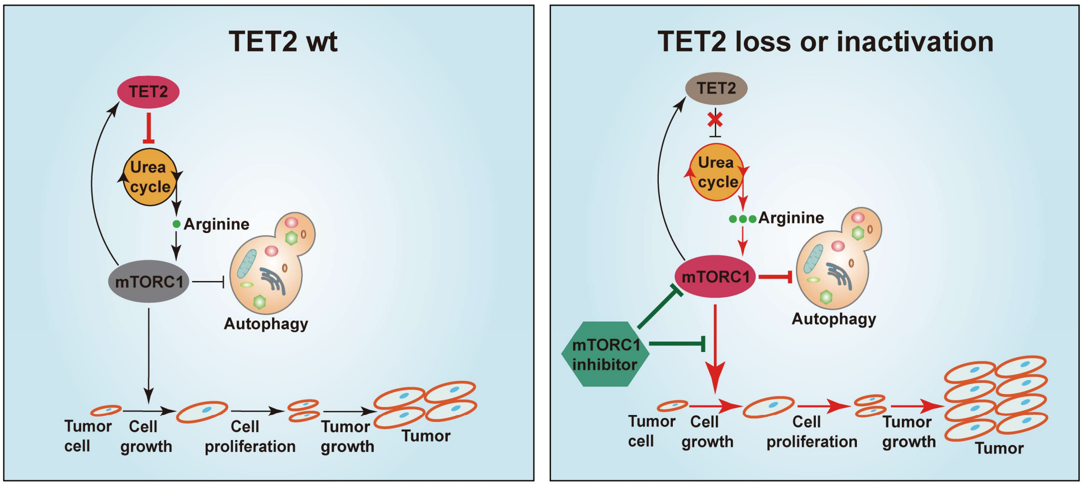

Supplementary Fig. S7 TET2 suppresses mTORC1 signaling through urea cycle by mRNA oxidation to inhibit tumor cell growth and promotes autophagy. In TET2 wild type tumors, TET2 suppresses mTORC1 signaling through urea cycle by mRNA oxidation to inhibit tumor cell growth and promotes autophagy (left panel). In TET2 loss or inactivation tumors, urea cycle is activated and arginine production is increased, which in turn activates mTORC1 signaling to promote cell growth, and then cell proliferation, resulting in eventual tumor growth, which can be targeted by suppression of mTORC1 with its inhibitors (right panel). Black and red lines mean weak and strong activation (or inhibition), respectively.

Supplementary Table S1. qPCR primers for human RNA

Name	Sequence (5’-3’)
ACTIN-F	CATGTACGTTGCTATCCAGGC
ACTIN-R	CTCCTTAATGTCACGCACGAT
ARG1-F	TGGACAGACTAGGAATTGGCA
ARG1-R	CCAGTCCGTCAACATCAAAACT
ASL-F	GCCGAGATGGACCAGATACTC
ASL-R	CTGCCGTTGCACCAATGAG
ASS1-F	TCCGTGGTTCTGGCCTACA
ASS1-R	GGCTTCCTCGAAGTCTTCCTT
CPS1-F	AATGAGGTGGGCTTAAAGCAAG
CPS1-R	AGTTCCACTCCACAGTTCAGA
OTC-F	TAGCTCTCTGAAAGGTCTTACCC
OTC-R	AGGTGCATTCCGAATTTCGCT
TET2-F	CAAGGCTGAGGGACGAGAAC
TET2-R	ATCCACAAGGCTGCCCTCTA
GAPDH-F	GGAGCGAGATCCCTCCAAAAT
GAPDH-R	GGCTGTTGTCATACTTCTCATGG
TNS4-F	AGGACACCAGAACTCCGTTCA
TNS4-R	TCTCGGGTGATGTTTGGCTTA
ALB-F	TGCAACTCTTCGTGAAACCTATG
ALB-R	ACATCAACCTCTGGTCTCACC

Supplementary Table S2. qPCR primers for mouse RNA

Name	Sequence (5’-3’)
mActin-F	GGCACCACACCTTCTACAATG
mActin-R	GGGGTGTTGAAGGTCTCAAAC
mArg1-F	CCACAGTCTGGCAGTTGGAAG
mArg1-R	GGTTGTCAGGGGAGTGTTGATG
mAsl-F	GGGAAGCTACACACAGGACG
mAsl-R	GCTGAGCTCTCTGCAAGTGT
mAss1-F	GCCAAGTGTACATCCTCGGT
mAss1-R	GACCTTGCTCTGAAGGCGAT
mCps1-F	CGGGAAGTAGAGATGGACGC
mCps1-R	CCTTGGCTGATGGTCTGTGT
mOtc-F	GCTAGCAGAGCAGTATGCCA
mOtc-R	ATACATTGCCTCCACGTGCT

Supplementary Table S3. ARG1 primers for TET2 RIP

Name	Sequence (5’-3’)
P1-F	GTCACTGAGGGTTGACTGACTG
P1-R	GTCATTAGGGATGTCAGCAAAGG
P2-F	CCTTTCAAATTGTGAAGAATCCAAGGT

P2-R	GCAAGTTTCCACTTGTGGTTGT
P3-F	GGACAACCTGTATCTTTCCTCCTG
P3-R	CTAGTAGATAGCTGAGTGTTTCTTCCATC
P4-F	TGTGGAAACATCCGATATAAATCTCATAGT
P4-R	GTTAGAATTTCCACATCTTGAATTTTACACC
P5-F	GCTTATATTTTCTAACTTGGCAAAAGACTT
P5-R	CACATTTTTTGAATGACATGGACACATAG

Supplementary Table S4. ASL primers for TET2 RIP

Name	Sequence (5'-3')
P1-F	GCGACACTATCCGTGCGG
P1-R	GCTGTAGGCTTTGCTGCCT
P2-F	CTCCTCACCAAGGCCGAG
P2-R	CAGCCTGAGGTCTGTGACC
P3-F	GATGCGGCAGACCTGCT
P3-R	GTCTCGGGTCAGTGCCAC
P4-F	CTGAGCGGCTGCTGGAG
P4-R	CAGGAACTCGGCCACAAAGT
P5-F	GCTTCGCTGTGCATGACC
P5-R	GGTCATCAGGAGCCCGG
P6-F	GAAGTGTCAGACACTATGAGTGCC
P6-R	CAGCTCCTGCAGTGACAGC
P7-F	TCGACTGGCAGATCCGC
P7-R	CCAAAGTGCTGGGATTACAGG
P8-F	GCAAGGTGCGAGGATGC
P8-R	GTTATCCAGGCTGGAATGCAG
P9-F	GCGGAGAAAACTGGGCAAG
P9-R	GTCTTCCTGTTGCCCAGGC

Supplementary Table S5. ASS1 primers for TET2 RIP

Name	Sequence (5'-3')
P1-F	CATCTGCAGGTGGCTGTGA
P1-R	GTGAACCACTCGGGCTCG
P2-F	GGAACTCACGCCTCCAATCC
P2-R	CCAAGCTTCAGTGCCTTCTTCC
P3-F	GGAGTTTGTGGAGGAGTTCATCT
P3-R	CCAGTGAGTAGCAGCTGAGC
P4-F	GAGGATGCCTGAATTCTACAACCG
P4-R	GGCTTTGGCTGGGTCCTG
P5-F	GTCCCTGTGAAGGTGACCAA
P5-R	TGGTGAAGGCCTCGATGTC
P6-F	GCACAGCCCTGAGTGTGA
P6-R	TTCCTTCAGCCTGAGGGAATT

P7-F	CTCCTCAATTTGCAGATCCCC
P7-R	GATGACAACGTTTGCAGGCT

Supplementary Table S6. CPS1 primers for TET2 RIP

Name	Sequence (5'-3')
P1-F	TGTAGTTGCTTTCTTAACCTCATCAAATTC
P1-R	TGTGTGCTGTCTGTGCCTT
P2-F	GCTTAAAGCAAGCGGATACTGTC
P2-R	TGCCTGTCTTCCGTAGCC
P3-F	GATGGCTTCCACTGGAGAGG
P3-R	GACATTGTTGGCGTTGAGCC
P4-F	GATGCAGACACCCCAGCC
P4-R	GACTGTAAGGTTTTGAAGAAGTGACTG
P5-F	CCTAAGTTACTCTTCATGAGATTTCATCCA
P5-R	TAGAAAGAGTTCAGAAAATAGAACAGCAAC
P6-F	CACTATCTGCAAACTCAGGACACT
P6-R	CTTCAATATAATGGGAAGGGATAACAATATTCC
P7-F	GAGAAATGTGACAGAGGCATTTAGAG
P7-R	GAAATCCATAGTCTAAAGCAGGAGAATC
P8-F	CTGGACTATATCAGCAGATGGTAGAC
P8-R	GGAATGAACCTTACTTCCAAGTTATTCC
P9-F	CTTAAGACGATGGATTCTGTTGAACTATG
P9-R	GTTCACAATTAGAGCACTTGTAATAACAGA

Supplementary Table S7. OTC primers for TET2 RIP

Name	Sequence (5'-3')
P1-F	CGCTGGCTAACTTGCTGTG
P1-R	GAAGGTCACGGCCCTTCA
P2-F	TATATGCTATGGCTATCAGCAGATCTG
P2-R	CACCCAAATGAATATCTTGTGTGGTAAG
P3-F	GTCTCACGGACACGGCC
P3-R	GAGCGTGAGGTAATCAGCCA
P4-F	CAGGAACACTATAGCTCTCTGAAAGG
P4-R	TCTCTTTGGCATACTGCTCTGC
P5-F	CCAAGCTGTTGCTGACAAATGAT
P5-R	CAGAGGCAGCAACTTTAGCAG
P6-F	CTGGACATTTTTACACTGCTTGCC
P6-R	TAGGCTTCTGGAGCTGAGGT
P7-F	TGTTGTGTTACTTGTCAAGAAAGAAGC
P7-R	GGCTTAAGGAAAGTTTCACAATGGC
P8-F	GTGCTGATGCACTGTAATACGT
P8-R	GGTAATAAGCATAGATTACACTTAATGGCTT

Supplementary Table S8. qPCR primers for 5mC/5hmC/YBX1/HuR RIP

Name	Sequence (5'-3')
ARG1-F	TGTGGAAACATCCGATATAAATCTCATAGT
ARG1-R	GTTAGAATTTCCACATCTTGAATTTTACACC
ASL-F	GCAAGGTGCGAGGATGC
ASL-R	GTTATCCAGGCTGGAATGCAG
ASS1-F	CTCCTCAATTTGCAGATCCCC
ASS1-R	GATGACAACGTTTGCAGGCT
CPS1-F	CCTAAGTTACTCTTCATGAGATTTCATCCA
CPS1-R	TAGAAAGAGTTCAGAAAATAGAACAGCAAC
OTC-F	CTGGACATTTTTACACTGCTTGCC
OTC-R	TAGGCTTCTGGAGCTGAGGT
